# Integrative Transcriptomic Analysis Reveals a Multiphasic Epithelial–Mesenchymal Spectrum in Cancer and Non-tumorigenic Cells

**DOI:** 10.3389/fonc.2019.01479

**Published:** 2020-01-22

**Authors:** Nicholas Panchy, Cassandra Azeredo-Tseng, Michael Luo, Natalie Randall, Tian Hong

**Affiliations:** ^1^Department of Biochemistry & Cellular and Molecular Biology, The University of Tennessee, Knoxville, Knoxville, TN, United States; ^2^National Institute for Mathematical and Biological Synthesis, Knoxville, TN, United States; ^3^Department of Biochemistry, New College of Florida, Sarasota, FL, United States; ^4^Department of Applied Mathematics, New College of Florida, Sarasota, FL, United States; ^5^Department of Mathematics & Statistics, The College of New Jersey, Ewing Township, NJ, United States; ^6^Department of Mathematics and Computer Science, Austin College, Sherman, TX, United States

**Keywords:** hybrid EMT states, ZEB1, breast cancer, cell migration and proliferation, tumor cell heterogeneity

## Abstract

Epithelial–mesenchymal transition (EMT), the conversion between rigid epithelial cells and motile mesenchymal cells, is a reversible cellular process involved in tumorigenesis, metastasis, and chemoresistance. Numerous studies have found that several types of tumor cells show a high degree of cell-to-cell heterogeneity in terms of their gene expression signatures and cellular phenotypes related to EMT. Recently, the prevalence and importance of partial or intermediate EMT states have been reported. It is unclear, however, whether there is a general pattern of cancer cell distribution in terms of the overall expression of epithelial-related genes and mesenchymal-related genes, and how this distribution is related to EMT process in normal cells. In this study, we performed integrative transcriptomic analysis that combines cancer cell transcriptomes, time course data of EMT in non-tumorigenic epithelial cells, and epithelial cells with perturbations of key EMT factors. Our statistical analysis shows that cancer cells are widely distributed in the EMT spectrum, and the majority of these cells can be described by an EMT path that connects the epithelial and the mesenchymal states via a hybrid expression region in which both epithelial genes and mesenchymal genes are highly expressed overall. We found that key patterns of this EMT path are observed in EMT progression in non-tumorigenic cells and that transcription factor ZEB1 plays a key role in defining this EMT path via diverse gene regulatory circuits connecting to epithelial genes. We performed Gene Set Variation Analysis to show that the cancer cells at hybrid EMT states also possess hybrid cellular phenotypes with both high migratory and high proliferative potentials. Our results reveal critical patterns of cancer cells in the EMT spectrum and their relationship to the EMT process in normal cells, and provide insights into the mechanistic basis of cancer cell heterogeneity and plasticity.

## Introduction

Epithelial–mesenchymal transition (EMT) is a fundamental cellular process in which rigid epithelial cells convert to motile mesenchymal forms. Canonical EMT and its reversal, mesenchymal–epithelial transition (MET), occur in embryogenesis, and they are critical for the formation of body plans and new organs in metazoans (1, 2). Numerous reports have shown that EMT is also activated during acquisition of a metastatic phenotype by tumor cells (3–5). In this scenario, EMT enables cells to migrate to distant organs or invade adjacent tissues, whereas MET allows cells to settle and proliferate (6). Inhibitions of EMT or MET have been shown to reduce the metastatic potentials of tumor cells (7–9). In addition, EMT was shown to promote chemoresistance (10, 11), suggesting the multifaceted roles of EMT in cancer progression and treatment.

Recent data and theoretical studies suggest a remarkable diversity of normal and cancer epithelial cells in terms of their E and M properties. Particularly, mathematical models and experiments show that partial forms of EMT give rise to intermediate (or hybrid, or transition) cellular phenotypes that exist between the extreme E and extreme M states, and that such phenotypes can be stable (12–18). These intermediate phenotypes were observed in non-tumorigenic epithelial cells during the EMT process induced by extracellular stimuli, such as transforming growth factor beta (TGF-β) (19). While the precise roles of EMT and its associated cellular states in cancer progression may be complex, recent single-cell transcriptomic analysis showed that various tumor cells are enriched with intermediate EMT cellular phenotypes (20), suggesting the prevalence of such cell states during cancer progression. In addition, previous survival analysis has shown that the intermediate EMT states are associated with poor prognosis of breast cancer patients (21, 22). Together, these studies suggest the importance of understanding an EMT spectrum that contains intermediate cell states in cancer cells.

During EMT, cellular properties such as adhesion, motility, and proliferation are altered dramatically through the coordination of two major molecular programs (E and M). Previous transcriptomic studies showed that several hundred epithelial-related genes (E-genes) and mesenchymal-related genes (M-genes) are down-regulated and up-regulated, respectively (14, 23–25). Previously, one-dimensional EMT spectrums were used to describe the cellular diversity in EMT (14, 26). This approach is useful to understand multiple EMT phenotypes in a concise manner. However, given the complexity and importance of E- and M-gene coordination, the one-dimensional spectrums do not provide a complete view of EMT process and its associated cellular diversity. In more recent studies, landscapes of cellular states in both E-gene activity and M-gene activity are used to describe cancer cell transcriptomes and specific perturbations leading to EMT/MET (27). Nonetheless, general patterns of cancer cell distributions in the two-dimensional EMT spectrum are unclear, and it is not known how such distributions are related to the EMT process in normal cells. In addition, it is not clear whether the multiple steps of EMT marked by the intermediate EMT state(s) involve the same degree of the coordination between E and M programs.

In this study, we performed integrative transcriptomic analysis that combines cancer cell transcriptomes, time course data during EMT induction, and transcriptomic changes upon perturbations of EMT factors. We systematically characterized the distributions of cancer and non-tumorigenic cells in terms of their E-gene and M-gene activities with Gene Set Variation Analysis (GSVA) and statistical models. We found that there is a significant diversity of cancer cells in their E-gene and M-gene activities, which cannot be described by an EMT spectrum that assumes linear coordination between E and M programs. We identified a non-linear EMT path that connects E and M, and hybrid cell states can be used to describe a large fraction of cancer cells in multiple organs. Notably, this EMT path involves a region in which the activities of E-genes and M-genes are both relatively high, and the pattern of this path is consistent with the EMT process in non-tumorigenic epithelial cells with respect to time. We identified key regulators that may contribute the multiphasic EMT that is elucidated by the EMT path. We found that the hybrid EMT gene expression region also corresponds to cell states with hybrid cellular phenotypes, including high motility and proliferative potentials. Together, our analyses characterized the multiphasic nature of EMT in a comprehensive and quantitative manner, and elucidated the connection between the diversity of cancer cells and the normal EMT progression in gene expression space, suggesting that multiple attractors in EMT are an intrinsic property that is reflected in both cancer and normal cells.

## Methods

### Transcriptomic Data

RNA-seq-based gene expression data were obtained for 1,215 invasive breast carcinoma samples (BRCA), 183 pancreas adenocarcinoma samples (PAAD), 576 lung adenocarcinoma samples (LUAD), 309 cervical squamous cell carcinoma and endocervical adenocarcinoma samples (CESC), and 550 prostate adenocarcinoma samples (PRAD) from The Cancer Genome Atlas (TCGA) using the R package “TCGABiolinks” (28, 29). These data were pre-processed via upper quartile normalization of RNA-Seq by Expectation Maximization (RSEM). Previous studies using TCGA transcriptome data have elucidated key factors contributing to cancer cell plasticity (24, 30, 31). As a control for non-EMT cancer cell types, we also obtained 173 acute myeloid leukemia cell samples (LAML) from TCGA. Time course transcriptomic data for TGF-β-treated MCF10A cells were obtained from a recent study by Zhang et al. (32). Transcriptome data for combinatorial perturbations of TGF-β and ZEB1 in MCF10A cells were obtained from a recent study by Watanabe et al. (23). Both of the Zhang et al. and Watanabe et al. studies employed RNA-Seq data and normalized the results as fragments per kilobase million (FPKM).

### E and M Scores

**Figure 1:**
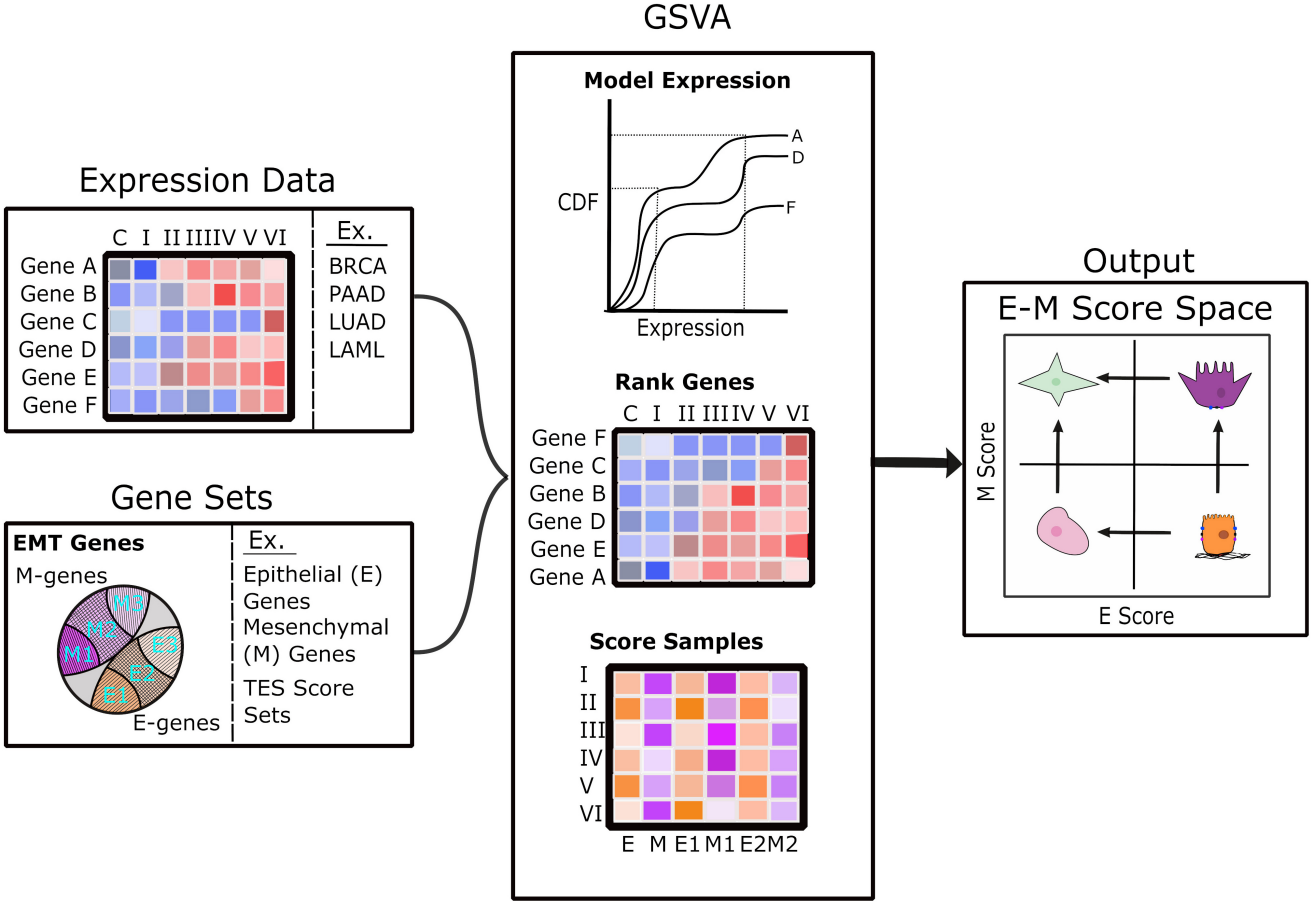
Diagram of the analysis pipeline to map transcriptome samples to E–M score space. The left panels show the scoring pipeline including transcriptomic data (top) and various gene sets (bottom). The center panels show a brief description of the GSVA scoring process that includes modeling gene expression as a cumulative distribution function, ranking of genes across samples, and the estimation of scores using Kolmogorov–Smirnov statistics. The right panel shows E–M score space annotated with possible cell states in each quadrant: terminal-E (lower right), terminal-M (upper left), hybrid intermediate (upper right), and characterless intermediate (lower left).

We computed scores for E and M scores with GSVA (33). For each sample (one transcriptome), we used a list of E-genes and a list of M-genes from Tan et al. (26) as two signature gene sets to compute the two scores, respectively. Briefly, GSVA estimates a cumulative density function for each gene using all samples, ranks genes across samples, and then calculates a score between −1 and 1 for each gene set using the Kolmogorov–Smirnov random walk statistic. GSVA scoring was implemented using the R package “GSVA” (33). The scoring procedure is illustrated in Figure 1.

### Gaussian Mixture Model

We built Gaussian Mixture Models (GMMs) for cancer cell transcriptomes in terms of their E and M scores to infer the subpopulations in the EMT spectrum. We tested one to nine subpopulations, and we used Bayesian Information Criteria to select the optimal number of subpopulations (Supplementary Tables 1–5). For each number of subpopulations, we tested six different models based on various assumptions on covariance [excluding those that allowed for non-diagonal or cluster-specific relationships among E and M scores between models (34)]. Among the models with different numbers of subpopulations, we selected the best subpopulation number using the best score based on Bayesian Information Criterion (BIC). We found that the five-cluster models had the lowest BIC scores in terms of both mean score in covariance models and the minimum score (Supplementary Figure 1). We therefore selected five-cluster models for the subsequent analysis. To select the most representative model with a particular assumption of covariance from the models with the best subpopulation number, we compared the distributions of all candidate models and selected those with the most consistent distribution across all models. We found that the covariance assumptions EII, EEI, VEI, and VVI (34) generated the most robust models within data sets, except for the LUAD data where there was a significant difference between equal volume models (EII, EEI) and unequal variance (VEI and VVI) (Supplementary Figure 2). While the unequal variance models had slightly better BIC, model predictions overall had higher uncertainty values (Supplementary Figure 3). As such, we chose to exclude unequal volume models and used EII for clustering transcriptome data except BRCA, where it was outscored by EEI but gave similar results. GMMs were implemented using the “mclust” package in R (34). To show that our main conclusions are not sensitive to the choice of the five-cluster models, we performed additional analysis with four-cluster and six-cluster models, which had BIC scores moderately higher than those with five-cluster models (Supplementary Figure 1). Although our main analysis focused on five-cluster models, additional comparisons of these models were performed, and they are described in later sections.

### Segmented Regression

To infer the one-dimensional EMT spectrums (EMT paths) from the two-dimensional scores, we used segmented regression models (35). For cancer cells with ample amount of data and significant heterogeneity of multiple possible EMT paths, the models were based on consecutive sample clusters that can be sequentially ordered in the E–M space by adjacency, and the assumption that paths must proceed from the most extreme E to the most extreme M state by passing through the minimum number of clusters (i.e., four clusters in BRCA, three clusters in all others). In the regression model, we chose the independent variable to be the projection of a pair of E–M scores onto a straight line crossing with the origin with slope of −1, representing a hypothetical linear progression of EMT, and the dependent variable to be the projection onto its orthogonal line, representing the deviation from the linear EMT progression. For time course TGF-β-driven EMT data with the time labels as the unambiguous independent variable, all data points were used to infer two models (E and M scores as functions of time). In all models, piecewise relationships between E and M scores were obtained. To test the existence of non-zero difference in slope parameter, we employed the Davies' test and a pseudo Score statistic test with a null hypothesis that there is a zero difference in slopes (36–38). Models with one to four breakpoints were then tested, and the respective maximum adjusted *R*^2^ values were used to select the best model (see Supplementary Table 6). For time course data, we used the E score and M scores relative to time to estimate breakpoints between 2 and 3 days (stating value = 2.5 days) and between 8 and 12 days (stating value = 10 days) for both E and M, respectively, and compared them to models with only one breakpoint. We found that the E scores only supported a segmented breakpoint when both breakpoints were used, while the two-breakpoint M score model outperformed a model with one breakpoint, which was always placed around 8 days (adjusted *R*^2^, 0.941 vs. 0.939). The approach we use to estimate breakpoint positions calculate a 95% confidence interval using a score-based approach that accounts for the non-differentiable, non-concave nature of likelihood function for breakpoints (35, 39) and we found that the confidence interval of the first E break point (0–4.6 days) covered the most sampled time points and therefore had the greatest impact on the time course model. Therefore, to find the upper and lower bound for the time course model, we modified the model by placing the first E break point at the extremes of the confidence interval (i.e., 0 days and 4.6 days). Comparably, the confidence interval of other breakpoints was either too small (0–1.25 days for the first M) or the range was between time samples (i.e., between 8 and 12 days for the second E and M breakpoint), such that varying them had little impact on the model as they did not alter the division of samples into different segments. Segmented models and analysis were implemented using the “segmented” package in R (39).

### Differences in TES Scores and EMT Factors Expression Between Clusters

Differences in TES scores and EMT gene expression between clusters were assessed using Welch's *t*-test, and the resulting *p*-values were adjusted using Benjamini–Hochberg correction for multiple hypothesis testing. Expression of individual genes in TCGA data sets was measured using normalized RSEM values derived from the TCGA processing pipeline (40).

### Clustering of E- and M-Genes

To infer clusters of E- and M-genes controlled by different regulatory circuits that connect to TGF-β and ZEB1, we employed a semi-supervised learning method that we used in our previous study. Briefly, EMT genes were clustered using a transcriptome data set obtained from combinatorially perturbed MCF10A cells with up- or down-regulation of TGF-β and ZEB1 (23). This unsupervised clustering was achieved by using a Self-Organizing Maps (SOM) algorithm on a 10 × 10 grid (41, 42). Each node was classified by counting the number of previously annotated E or M genes and computing their ratios (this supervised step is essentially a *k*-nearest-neighbors algorithm). To obtain gene clusters among E-gene nodes and M-gene nodes, respectively, we used the hierarchical clustering method and we selected the optimal numbers of clusters (four E-gene clusters and four M-gene clusters) using the within-cluster sums of squares error (the elbow criterion). Tables of EMT related scores and cluster classifications for samples in TCGA and EMT time course are available in Supplementary Files 1, 2, respectively.

### GSVA for Phenotypic Inference

To infer phenotypic changes when cells move along the EMT path, we employed GSVA in a way similar to what we used for computing E and M scores. We focused specifically on cancer samples for the BRCA data set, because the presence of normal tissue samples had a significant effect on functional scoring, specifically with respect to migration (see Supplementary Figure 4). We obtained curated gene sets from the Broad Institute and selected four representative gene sets each for cell migration and for cell proliferation to quantify the phenotypic enrichment of the corresponding cellular properties in each subpopulation along the EMT paths (for a full description of gene sets, see Supplementary Table 7) (43). Differences in score between clusters were assessed using the same test and correction procedure as EMT factors. To exclude the possibility that gene sets carry redundant information with the E- and M-genes, we calculated the percentages of overlapping genes between the phenotypic and pathway gene sets and the EMT gene sets, and in no case did more than 20% of genes in a phenotypic set belong to the E-gene set or M-gene set.

## Results

### Multimodal Distribution of Cancer Cells in the EMT Spectrum

**Figure 2:**
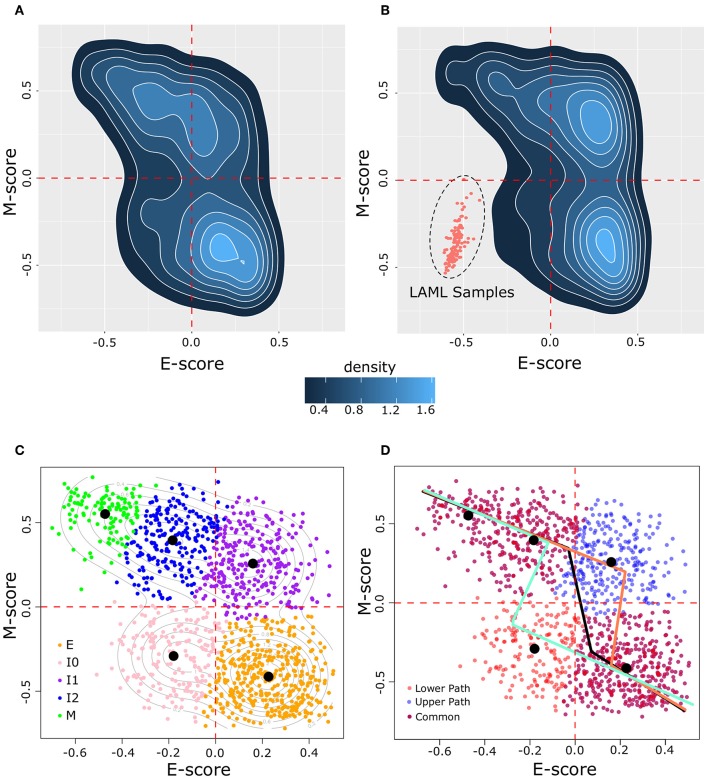
Distribution of BRCA samples in E–M score space following GSVA scoring. **(A)** Density of BRCA samples in E–M score space. Lighter blue color indicates higher density and darker blue indicates lower density. **(B)** Density of samples in a merged BRCA-LAML data set demonstrating the positioning of samples of a non-epithelial origin in the lower left (low-E–low-M) quadrant beyond the distribution of BRCA samples. **(C)** Clustering of BRCA samples by GMM. Individual samples are indicated by points in E–M score space with their assigned cluster indicated by color (E = orange, I0 = pink, I1 = purple, I2 = blue, M = green). Contour lines indicate the predicted distribution of the underlying models. Black dots denote the center of each Gaussian distribution. **(D)** Segmented models E–M score relationship among BRCA samples. Three models are shown: one based on all BRCA samples (black line), one excluding I0 samples (upper path, light red line), and one excluding I1 samples (lower path, light blue line). Individual samples are shown by points with the color corresponding to whether the point is unique to the upper path (blue) or to the lower path (red), or common to both paths (purple).

To examine how breast cancer cells are distributed in terms of the degree of EMT, we obtained 1,215 samples from the TCGA breast cancer project (BRCA), which focuses on invasive carcinomas, and we computed a pair of scores that summarize the overall transcriptional activities of E- and M-genes for each cancer cell sample, respectively. These scores are based on GSVA using a list of 228 E-genes and a list of 188 M-genes as signature gene sets (26). We found that the BRCA sample transcriptomes are widely distributed across the space of E and M scores (Figure 2A). In particular, all four quadrants of the E–M score space contain at least 10% of the samples, reflecting the heterogeneity of cancer cells in terms of the degree of EMT. Nonetheless, the quadrant corresponding to low-E–low-M gene expression contains fewest samples with 187 (15.4%), whereas the high-E–high-M quadrant contains 248 (20.4%) samples. To exclude the possibility that the wide distribution and the low density of samples in the low-E–low-M region is due to the normalization in the scoring scheme, we combined these breast cancer samples with 173 acute myeloid leukemia samples (LAML), which have hematopoietic lineage origins that are distant from that of epithelial cells. We found that the distribution of BRCA samples is consistent with the previous results even in the presence of LAML samples (Figure 2B). BRCA samples remain widely distributed in high-E and/or high-M quadrants with fewer samples in the low-E–low-M quadrant that are close to but do not overlap with LAML samples. We found that the distribution of the M scores is wider than that of the E scores (Figure 2). This is consistent with a previous observation that M-gene expression is more divergent than E-gene expression during EMT (23).

We next asked how many subpopulations these breast cancer samples may contain in terms of the degree of EMT. We built a series of Gaussian Mixture Models (GMMs) based on the E and M scores of these samples. We evaluated these models using BIC, the consistency of clusters across different variance assumptions, and the number of cells that can be assigned to subpopulations (clusters) with high confidence (see Methods). We found that a five-cluster model best describes the overall distribution of BRCA samples. Our GMM model of BRCA (Figure 2C) includes an extreme E cluster (orange), an extreme M cluster (green), and three intermediate clusters (I0, I1, and I2, which are pink, purple, and blue, respectively). The I0 cluster contains most (178 of 187) samples that are in the low-E–low-M region, whereas the I1 and I2 clusters contain all (248 of 248) samples with the high-E–high-M expression profiles. Most of these samples (237 of 248) belong to the I1 clusters, with I2 lying along the border between high-E–high-M and low-E–high-M. To exclude the possibility that our conclusions are sensitive to the choice of number of clusters, we analyzed the GMMs for four and six clusters and we found that they had distributions of clusters similar to that of the five-cluster model (Supplementary Figure 5).

Although EMT is a process involving gene expression changes in high-dimensional space, it is useful to construct one-dimensional EMT spectrums (paths) to quantify the degree of EMT. Moreover, if these one-dimensional spectrums can be mapped to the overall E- and M-gene activities, one can further infer the changes of the (anti-)correlation between E- and M-gene activities during EMT/MET. Therefore, we constructed models that describe possible EMT spectrums quantitatively. We assumed that a path will connect E and M states by joining neighboring clusters as cells go from the extreme E-state and to the extreme M-state. We also assumed that paths will take the fewest possible steps between neighboring clusters (loops and backtracking were not considered). With these assumptions, two possible three-step paths of EMT can exist: E–I0–I2–M (the lower path) and E–I1–I2–M (the upper path). We built two piecewise linear models using segmented regression with data from these two sets of clusters (see Methods). Our assumption concerning EMT progression is supported by a non-linear relationship between the E and M score (Davies's test, *p* = 8.6 × 10^−3^) and the possible existence of a breakpoint in the relationship between E and M along each path (pseudo Score stat, *p* = 3.7 × 10^−8^ and *p* = 1.3 × 10^−3^ for upper and lower paths, respectively). Models for the upper (light red, Figure 2D) and lower (light blue, Figure 2D) paths had reasonable performance in fitting to their respective data points (*R*^2^ = 0.5 and 0.52 for upper and lower paths, respectively). The overall distribution of BRCA samples had an *R*^2^ of 0.29 with a single segmented model (black, Figure 2D).

Note that these paths do not necessary contain information about how cancer cells change their expression over time, because EMT and MET can occur one after the other at any stage of EMT/MET. Rather, they predict how the overall E- and M-gene activities are likely to change at any given state when cancer cells alter their expression profiles in an incremental fashion. These steady-state and transient changes may be triggered by changes of microenvironment or mutations in cancer cells.

We next asked whether the pattern of distributions of cancer cells in the E–M space is consistent across tumors from different organs. We obtained samples of pancreas (PAAD), cervical (CESC), prostate (PRAD), and lung carcinoma (LUAD) from TCGA, all of which were shown to involve EMT (8, 44–49). Samples from these cancers show similar distributions to that of the breast cancer samples (Supplementary Figure 6) and GMMs consistently generated four populations as the optimal models (Supplementary Figure 7) with rough correspondence to BRCA populations (excluding I2). Using the same approach that we applied to BRCA, we found that a segmented model with two distinct EMT paths fits better than a single segmented model in all cases (Supplementary Figure 8). Notably, the samples in the high-E–high-M states are the main population of the cells at the intermediate EMT states in PAAD (42 of 56, 75.0%) and PRAD (147 of 212, 69.3%). In contrast, high-E–high-M and low-E–low-M populations are comparable in LUAD (104 of 192, 54.1% low-E–low-M) and CESC (66 of 125, 52.8% low-E–low-M). As such, both the upper and lower paths could be possible routes of E/M variations in our cancer data.

### An EMT Path Involving a High-E–High-M State Revealed by Time Course EMT Data

**Figure 3:**
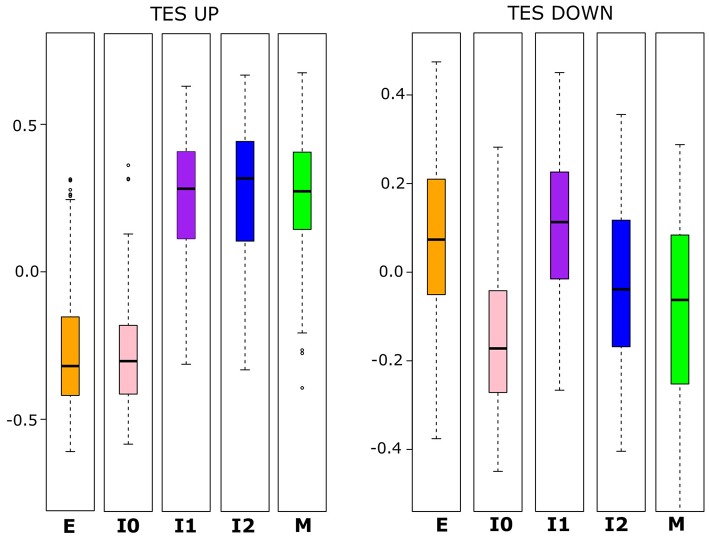
Boxplots showing the distribution of TES_UP (left) and TES_DOWN (right) scores that reflect the activating and repressing programs of TGF-β-induced EMT, respectively. Note that there is a significant difference (Welch's *t*-test, α < 0.05) in TES_UP between E and I1 and I0 and I2, but no significant difference among I1, I2, and M. For TES_DOWN, there is no significant difference between E and I1, but there are significant differences between E and I0, and between I1 and I2. I0 has a significantly lower TES_DOWN score than all other clusters do, but the magnitude of the difference is smaller between M and I0 than for E and I0. A full table of *p*-values and 95% confidence intervals can be found in Supplementary Table 8.

To further examine whether the variation in E and M scores among the populations of BRCA samples is driven by the canonical EMT (e.g., TGF-β induced) pathway, we applied GSVA to calculate TGFβ-EMT scores (TES) in order to track progression through EMT (24). This score has two components, one for genes that increase during TGF-β-induced EMT (TES_UP) and the other for genes that decrease during the same process (TES_DOWN). Importantly, while there is overlap between these gene sets and our E- and M-gene sets, the majority of both TES_UP (155, 81.7%) and TES_DOWN (70, 64.8%) genes are not used to define our E and M scores. With the distribution of these scores across the BRCA populations, we observed a pattern that follows our previous two-path model of EMT progression (Figure 3). TES_UP and TES_DOWN values were compared between each cluster using Welch's *t*-test; the *p*-values and 95% CIs for each comparison can be found in Supplementary Table 8. I0 samples have significantly decreased TES_DOWN relative to E samples (Welch's *t*-test, *p* = 1.97 × 10^−42^), consistent with a decrease in E-gene expression, while I1 has significantly increased TES_UP (Welch's *t*-test, *p* = 1.98 × 10^−132^), consistent with an increase in M-gene expression. As a consequence, transition from I1 to M involves a significant repression of E-gene activity, while transition from I0 to M involves a significant increase of M-gene activity along with a relatively small change of E-gene activity in terms of magnitude (lower bound = +0.015 E score), compared to the transition from I1 (lower bound = −0.217 E-score). Nevertheless, these results are consistent with an “upper path” of E–M variance where the initial changes result from EMT-driven activation of mesenchymal genes and a “lower path” of E–M variance where the initial changes result from EMT-driven repression of epithelial genes. These results suggest that canonical EMT is an underlying factor of E/M variation in our cancer samples. However, it remains unclear whether TGF-β-driven EMT in non-tumorigenic cells resembles either of the putative paths (24, 50).

**Figure 4:**
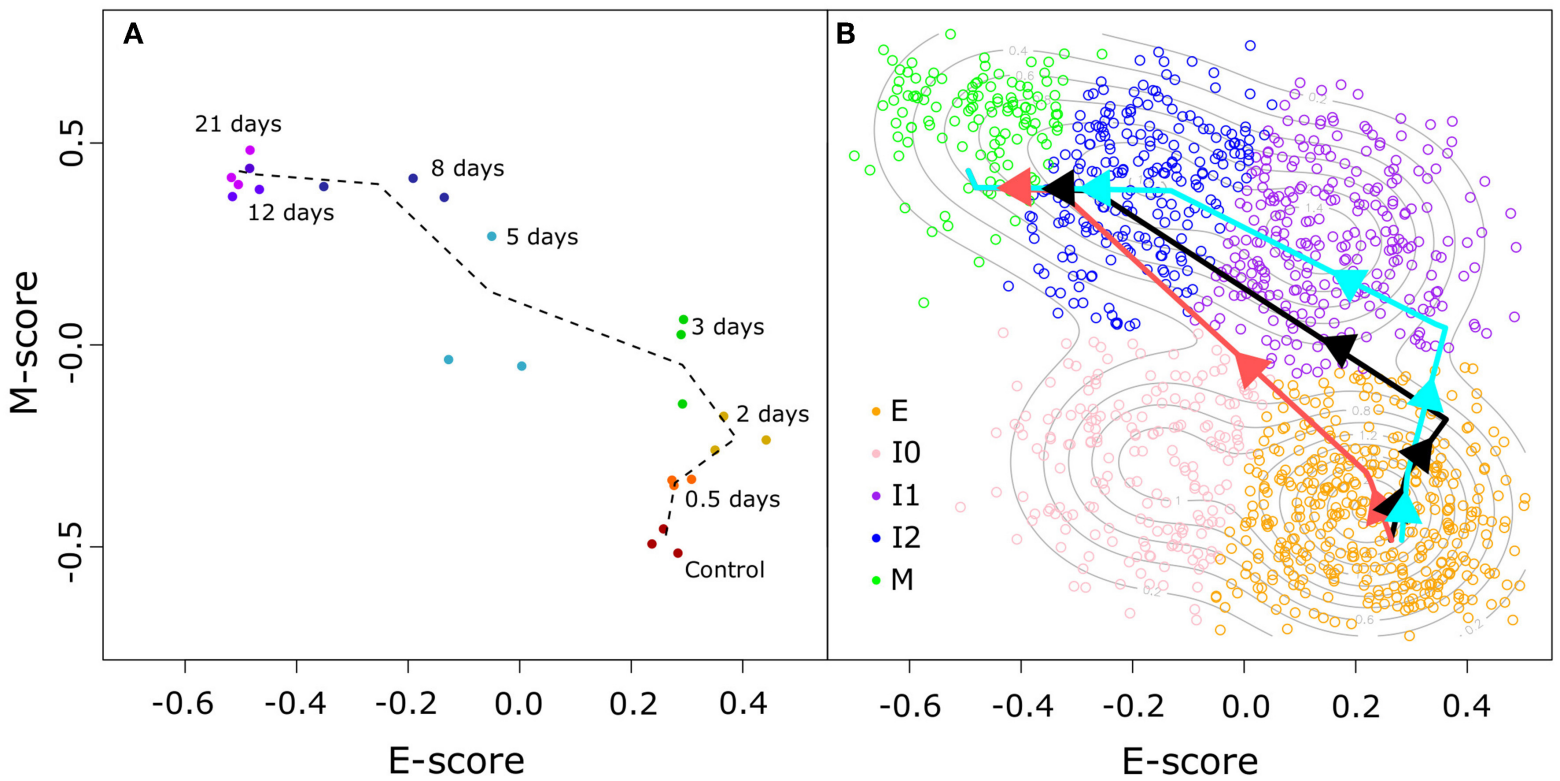
TGF-β-induced EMT time course in E–M score space. **(A)** Time course samples projected in E–M space. Progression of samples through time is indicated by color (from red to purple) and adjacent time points are linked by a dotted black line. **(B)** Segmented models of the relationship between the time since TGF-β induction of EMT and the E–M scores of treated cells. Different colored lines correspond to the best fit (black), lower bound (light red), and upper bound (light blue) models. Individual BRCA samples are indicated as open points with their color corresponding to the assigned cluster (E = orange, I0 = pink, I1 = purple, I2 = blue, M = green).

To address this, we used a time course transcriptome data set for MCF10A cells (non-tumorigenic human breast epithelial cells) treated with TGF-β (32) to compute E and M score for time points across EMT using the same procedure described earlier. The distribution of these time course data points in E–M score space (Figure 4A) shows that the TGF-β-driven EMT process involves an initial phase of significant increase of M-gene expression with a moderate change of E-gene activities, as well as a final phase of significant decrease of E-gene expression with a moderate change of M-gene activities. The middle phase of this process involves change of cell states near or within the high-E–high-M region. We next built a segmented regression model for all data points with time as the reference independent variable (see Methods). We found that the model generated a triphasic pattern with two break points with respect to both E and M scores (Figure 4B). Varying the placement of the first E score breakpoint was used to examine the upper (Figure 4B, light blue) and lower (Figure 4B, light red) bounds of the model of TGF-β-driven EMT, and in all cases, cell states primarily evolve through the high-E–high-M region when compared to our GMM models of BRCA samples (Supplementary Figure 9). Together with the EMT paths that we constructed for BRCA samples, our analyses show that the TGF-β-driven EMT primarily involves a path that crosses a high-E–high-M state, and this process is reflected in the distribution of a large fraction of cancer samples. These results imply that some intrinsic intermediate EMT attractors may govern both normal EMT process and a large number of cancer cells, and that a major population of cells at the intermediate EMT states may possess hybrid phenotypes in which the overall activities of E-genes and M-genes are both high.

### Divergence of EMT Genes in Multiphasic Transitions Regulated by Key EMT Factors

**Figure 5:**
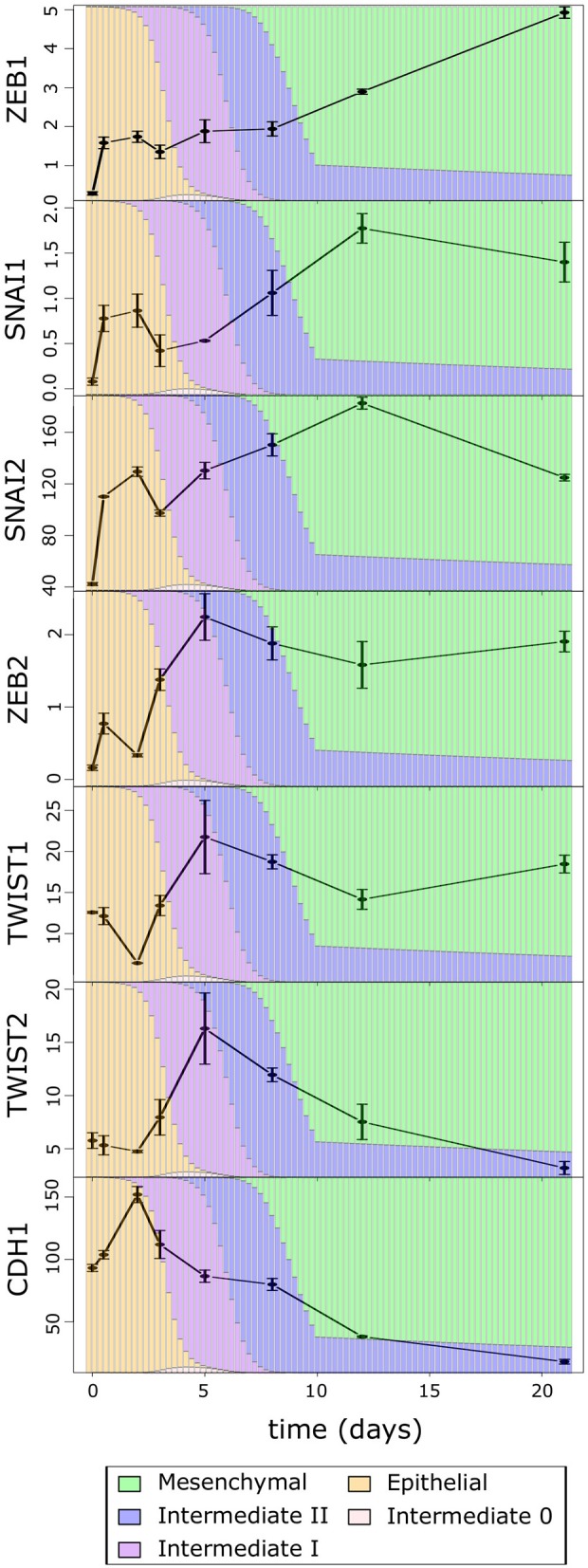
The average expression of EMT factors over the TGF-β-induced EMT time course. The color of the background bars in each plot indicates the predicated probability of samples at the point in time belonging to each cell state cluster. The probabilities were calculated by applying the BRCA GMM model to the segmented model of E–M scores with respect to time after TGF-β induction. Expression values are FPKM and bars indicate standard error of the mean.

We next asked which EMT factors may be responsible for the multiphasic EMT that we observed with the time course data as well as cancer cells. We first examined the time course expression of several core EMT promoting transcription factors, ZEB1/2, SNAIL1/2, and TWIST1/2 (Figure 5), which were shown to be critical for EMT and EMT-related physiological or pathological processes (51–57). Consistent with the pattern of overall M score, all these EMT factors show significant increase in the first phase (Day 0 to Day 5), although some (ZEB2, TWIST1, and TWIST2) only increase after a delay (Day 2), which is consistent with the transition into and through the high-E–high-M intermediates. Conversely, the late-phase expression of these factors is more divergent (Day 8 to Day 21): ZEB2 and TWIST1 do not show dramatic increase, while TWIST2 and SNAI1/2 decrease during the late-phase EMT. This is consistent with our analysis of EMT paths in BRCA samples where the increase in M scores becomes moderate after passing through the intermediate states. We found that the transcription factor ZEB1 showed robust increasing dynamics even in the late phase. Given the significant decrease in overall E-gene activity in late-phase EMT (e.g., E score and CDH1), the dynamical pattern of ZEB1 suggests its close association with the dynamics of many E-genes. In fact, in our recent perturbation analysis with MCF10A cells, we found that most of the annotated E-genes are down-regulated by ZEB1 in a causal fashion (23), and this is consistent with the dynamical anticorrelation that we found in the time course data.

**Figure 6:**
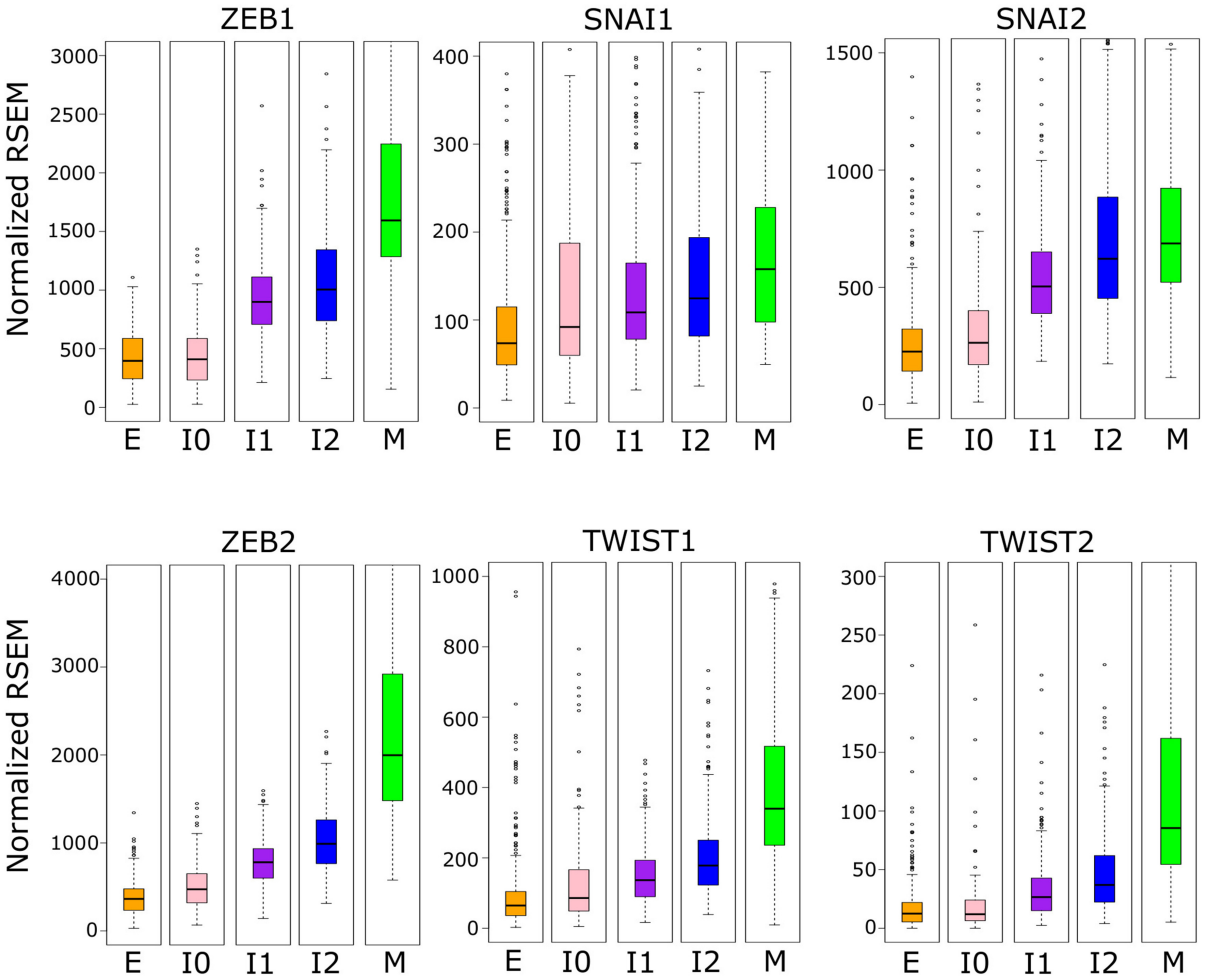
Boxplots showing the distribution of EMT factor expression across different clusters of BRCA samples indicated by color (E = orange, I0 = pink, I1 = purple, I2 = blue, M = green). Note that the difference between E–I1–I2–M clusters are significant for ZEB1, but SNAI1/2 show no significant difference between I2 and M, and TWIST1 is not significantly different between E and I1 but is between I2 and M. Expression values are normalized RSEM (RNA-Seq by Expectation Maximization) values (see Methods). A full table of *p*-values can be found in Supplementary Table 9.

We then asked whether the robust increase of ZEB1 also exists near the M-end of the EMT path in cancer cells, and we compared the distributions of ZEB1 expression across the five cancer cell clusters along the EMT paths (Figure 6). The *p*-values for comparing expression change between clusters by Welch's *t*-test can be found in Supplementary Table 9. Consistent with the time course EMT dynamics, ZEB1 showed an increase in each of the three steps of the E–I1–I2–M transition in BRCA (Welch's *t*-test, *p* = 3.97 × 10^−66^, 1.85 × 10^−4^, and 2.85 × 10^−15^, respectively). In contrast, SNAIL1/2 showed a significant increase from I1 to M (Welch's *t*-test, *p* = 3.84 × 10^−3^ and 4.96 × 10^−5^, respectively), but not I2 to M (Welch's *t*-test, *p* = 9.61 × 10^−2^ and 7.13 × 10^−2^, respectively), which reflects the long run decay of SNAI1/2 from their initial peak in M in the time course. We found that, in TWIST1, there is no significant difference between E and I1 (Welch's *t*-test, *p* = 1.43 × 10^−1^), consistent with the delay in the time course, but both TWIST1 and TWIST2 had a significant increase from I2 to M in BRCA samples (Welch's *t*-test, *p* = 5.93 × 10^−9^ and 7.39 × 10^−2^, respectively), a pattern not observed with time course data. In fact, TWIST1 and TWIST2 mirror ZEB1 in terms of their expression patterns in E, I1, I2, and M clusters. This dissimilarity between the cancer cells and the time course data was also observed for ZEB2, which showed the largest absolute change in mean between I2 and M. Consistent with the moderate increase of M score from E to I0, expression of ZEB1, TWIST1, and TWIST2 was not significantly different between I0 and E clusters (Welch's *t*-test, *p* = 2.66 × 10^−1^, 1.43 × 10^−1^, and 4.20 × 10^−1^ respectively). While the behavior of ZEB2 and TWIST1/2 was not consistent across data sets, unlike these EMT factors, ZEB1 has significant dynamical changes at the M-end of EMT path in both normal and cancer cells, indicating its primary role in robustly controlling E-gene expression at this phase of canonical EMT. On the other hand, the lack of ZEB1 variation in E–I0 suggests that if transition through low-E–low-M quadrant represents an alternative path of EMT, it accomplishes repression of E-genes through a ZEB1 independent pathway.

**Figure 7:**
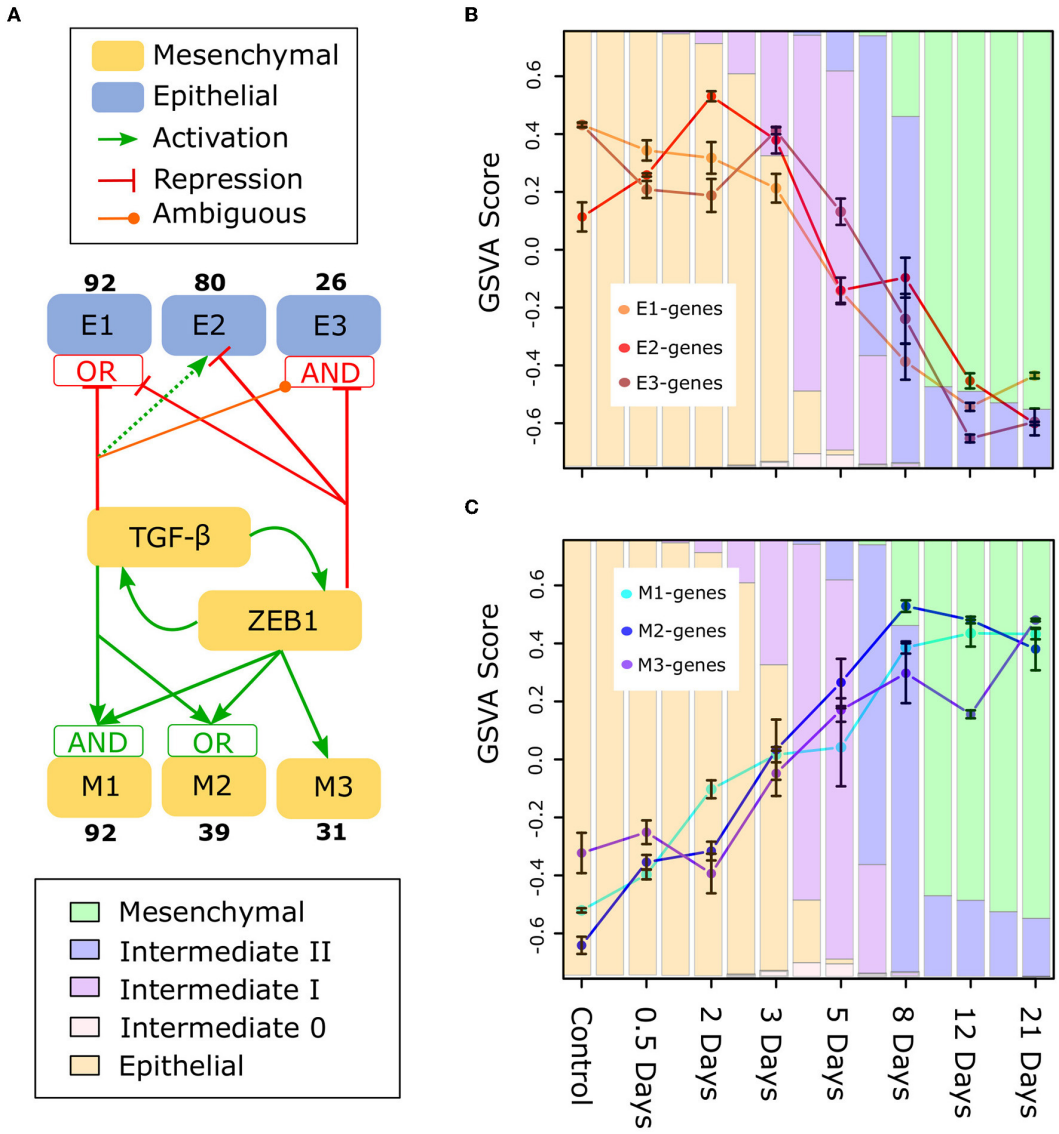
Subclusters of E and M genes in TGF-β induced EMT **(A)** A model of the regulation of subclusters of E (E1, E2, E3) and M (M1, M2, M3) genes by TGF-β and ZEB1 based on perturbation analysis (23). The boxes containing “AND” and “OR” indicate the type of logic gate integrating the regulatory signals of TGF-β and ZEB1: “OR” indicates that one factor can drive activation/repression independently, while “AND” indicates both are required. However, in the case of both “AND” gates, TGF-β alone can regulate the subcluster, either with reduced (M1) or with opposite (E3) affect (the latter is indicated by TGF-β being ambiguous for E3). Also, there is no gate integrating TGF-β and ZEB1 for E2 because ZEB1 suppresses activation by TGF-β when present (indicated by the dotted line for TGF-β). For the data and methodology underlying this regulatory model, see Supplementary Figure 10 for E-genes. **(B)** GSVA scores of E1, E2, and E3 subclusters across the TGF-β-induced EMT time course. **(C)** GSVA scores of M1, M2, and M3 subclusters across the TGF-β-induced EMT time course. The color of the background bars in each plot indicates the predicated probability of samples at the point in time belonging to each cell state cluster. The probabilities were calculated by applying the BRCA GMM model to the segmented model of E–M scores with respect to time after TGF-β induction. Bars that align with points on the line graph represent the predicated probabilities at the time point while columns between sample time points represented the predicated probabilities at the middle point of two neighboring time points (i.e., 0.25, 1.75, 2.5, etc.). Bars around each point indicate standard error of the mean.

To gain deeper understanding of the divergent expression patterns that contribute to the moderate change of E-gene expression in early-phase EMT, and/or to the moderate change of M-gene in late-phase EMT, we focused on gene clusters that are differentially controlled by TGF-β, a canonical EMT promoting factor important for metastasis (58), and ZEB1, a factor involved in regulation of most E-genes (23). Using a semi-supervised learning algorithm applied to a transcriptome data set for MCF10A cells that were combinatorially perturbed with up-/down-regulation of TGF-β and/or ZEB1 (23), we classified E- and M-genes into six major gene clusters [Figure 7A, Supplementary Figure 10, note that our previously analysis focused on M-gene clusters but not E-gene clusters (23)]. These gene clusters are regulated by TGF-β and ZEB1 with distinct circuits. Notably, the three E-gene clusters show a divergent expression pattern in early-phase EMT. In particular, the E2 cluster containing 80 E-genes show a significant increase from Day 0 to Day 3 (Figure 7B). The genes in this cluster are up-regulated by TGF-β via a ZEB-1-independent pathway and down-regulated by ZEB1, thereby forming an incoherent feed-forward loop. This network motif is likely responsible for the transient increase of this gene cluster around Day 3 as well as the overall increase in E score during the initial phase of EMT. Divergent expression patterns were also observed for M-genes: there is a significant difference between the M1/2 clusters and the M3 cluster, which is primarily regulated by ZEB1 (Figure 7C). M3 genes (e.g., TWIST1/2) do not exhibit increased expression until the intermediate phase of EMT progression, and this may be a general pattern of ZEB1 responsive M-genes. Furthermore, in contrast to M1 and M2, M3 still increases during Day 12 to Day 21, further reflecting the dynamics of ZEB1. Together, these results show that the heterogeneity of gene expression pattern contributes to the moderation of changes in E-gene expression during early-phase EMT and M-gene expression during late-phase EMT. The analysis further suggests that distinct gene regulatory circuits that connect EMT genes to ZEB1 and TGF-β are part of the mechanistic basis of such heterogeneity.

### Phenotypic Implications of Multiphasic EMT Spectrums

**Figure 8:**
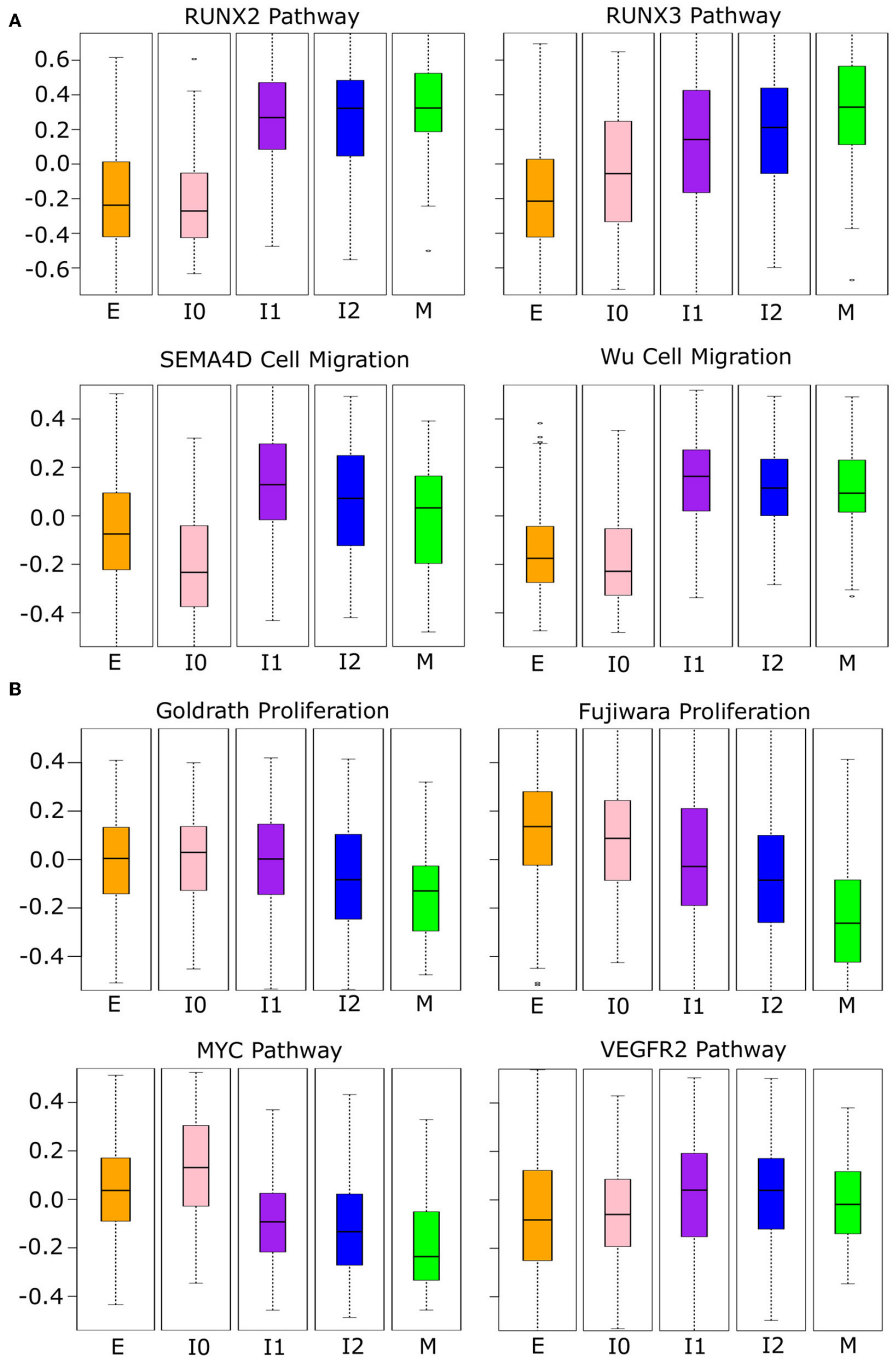
Phenotypic scores BRCA cancer samples. **(A)** Boxplots showing the distribution of migration-related gene set scores across different clusters of BRCA samples indicated by color (E = orange, I0 = pink, I1 = purple, I2 = blue, M = green). Note that, in all cases, there is a significant jump in score between E–I0 and I1–I2–M, except for RUNX3 where there is a constant, significant growth in scores from E to M. A full table of *p*-values can be found in Supplementary Table 10. **(B)** Boxplots showing the distribution of proliferation-related gene set scores across different clusters of BRCA samples indicated by color as in **(A)**. Note that proliferation scores of I1 samples are consistently higher than M samples and, in two cases, are comparable to that of E samples (Goldrath and VEGFR2). A full table of *p*-values can be found in Supplementary Table 11.

Since the intermediate cancer cell populations favor hybrid expression patterns with a high-E–high-M profile, we asked whether these cells possess hybrid cellular properties related to the EMT transcriptional program. We first performed GSVA with the breast cancer cell transcriptomes using several functional gene sets related to cell migration and proliferation from the Broad Institute GSEA database (see Methods). These two cellular properties are closely related to EMT and tumorigenesis, respectively. We used four curated cell migration gene sets including RUNX2, RUNX3, and SEMA4D-mediated pathways as well as a general migration gene set from Wu et al. (59) (Figure 8A). As cells progress from E-state to M-state, these migration-related genes are up-regulated significantly (Welch's *t*-test, see Supplementary Table 10). Remarkably, the I1 and I2 intermediate cell clusters show high activities of migration-related genes and their overall expression is comparable to that in the M state in all cases except for the RUNX3 pathway. Additionally, we observe a large increase in the expression of migration-related genes from E to I1, but not E to I0, except for the RUNX3 pathway, which shows a linear progression from E to M. We next applied GSVA to gene sets related to cell proliferation. Since there were no available gene sets for breast epithelial cells, we first used two gene sets describing the proliferation of other cell types: lymphocytes [Goldrath, (60)] and mice liver cells [Fujiwara, (61)]. In both cases (Figure 8B), as cells progress from E-state to M-state, proliferation-related genes are down-regulated significantly (Welch's *t*-test, see Supplementary Table 11), but I1 cells score significantly higher than M cells, and I1 is not significantly different from E with the Goldrath set. We observed the same pattern of proliferation driven by MYC pathway genes and a similar pattern among VEGFR2 pathway genes, though in the latter set, the proliferation of intermediate states is slightly higher than the extreme E state. We also observed that I0 has a higher proliferation score than all other clusters in the MYC pathway do, but in other proliferation sets, it is indistinguishable from E or is between E and I1. Taken together, our analysis of both proliferation and migration gene sets suggests that cancer cells in I1 and I2 may exhibit the same migratory potential of full-differentiated mesenchymal cells with only a partial reduction in proliferation compared to epithelial cells.

**Figure 9:**
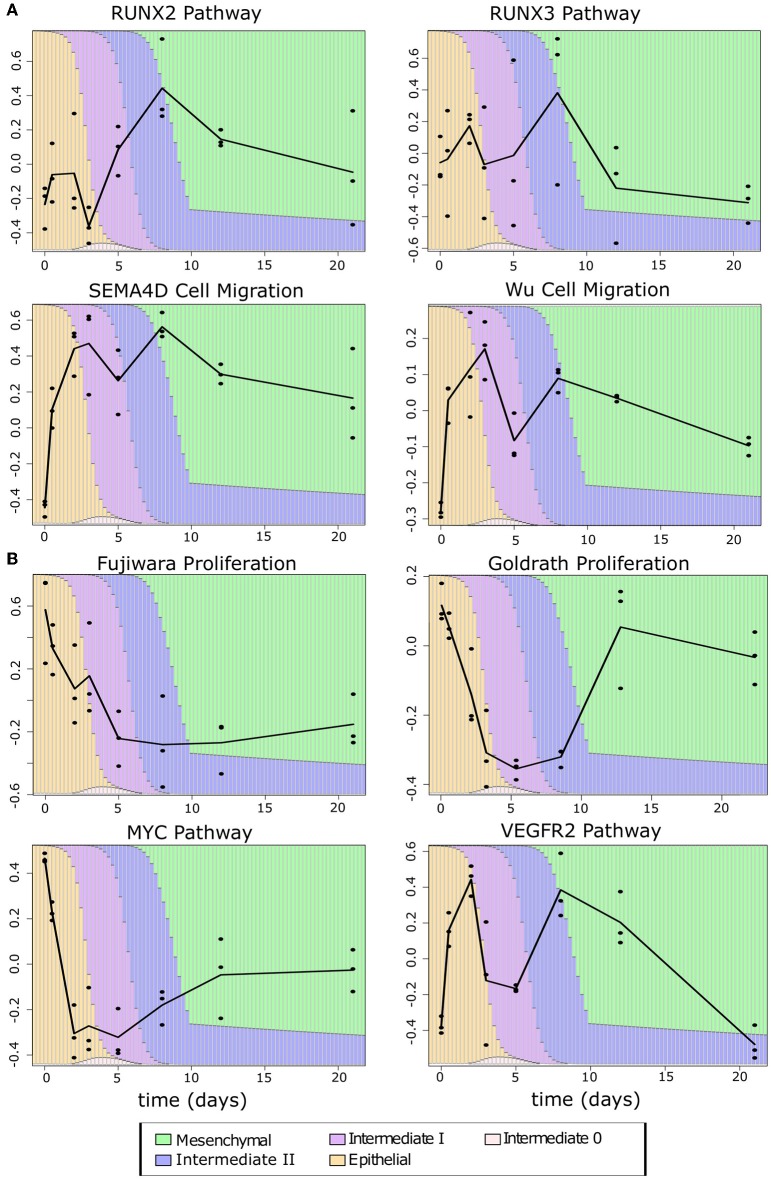
Phenotypic scores of EMT time course samples **(A)** Migration scores across the samples in the TGF-β-induced EMT time course. **(B)** Proliferation scores across the samples in the TGF-β-induced EMT time course. The color of the background bars in each line plot indicates the predicated probability of samples at the point in time belonging to each cell state cluster. The probabilities were calculated by applying the BRCA GMM model to the segmented model of E–M scores with respect to time after TGF-β induction. The black line indicates the change between sample averages at each experimental time point.

To exclude the possibility that the consistency of the observed pattern is simply due to overlap between the migration, proliferation, and E/M gene sets, we examined the number of overlapping genes between phenotypic gene sets, and between phenotypic gene sets and the EMT gene sets. The largest overlap occurs between the Wu gene set and E-gene (42, 22.9%) and M-genes (27, 14.7%). In the remaining pairwise comparisons of gene sets, there was either no or a small overlap (one to three genes) (Supplementary Table 12). This suggests that our phenotypic scores are generally independent from one another and from E–M scores, such that the functionally hybrid potentials of the I1 and I2 states are not likely due to an artifact in our method. Furthermore, this hybrid potential appears to be specific to cancer cells: the time course data showed the same pattern of an early gain of migration (Figure 9A), but only in the Fujiwara proliferation set did the intermediate EMT phase show high proliferation relative to the terminal mesenchymal states. In contrast, the intermediate EMT phase has lower proliferation capacity than either terminal E or M in scores obtained with the Goldrath set as well as the MYC and VEGFR2 pathways (Figure 9B), suggesting that the combined proliferative and migratory potential may be specific to cancerous intermediate states. Furthermore, this phenotypic combination may contribute to the “fitness” of the tumorigenic intermediate EMT states in metastasis (62).

**Figure 10:**
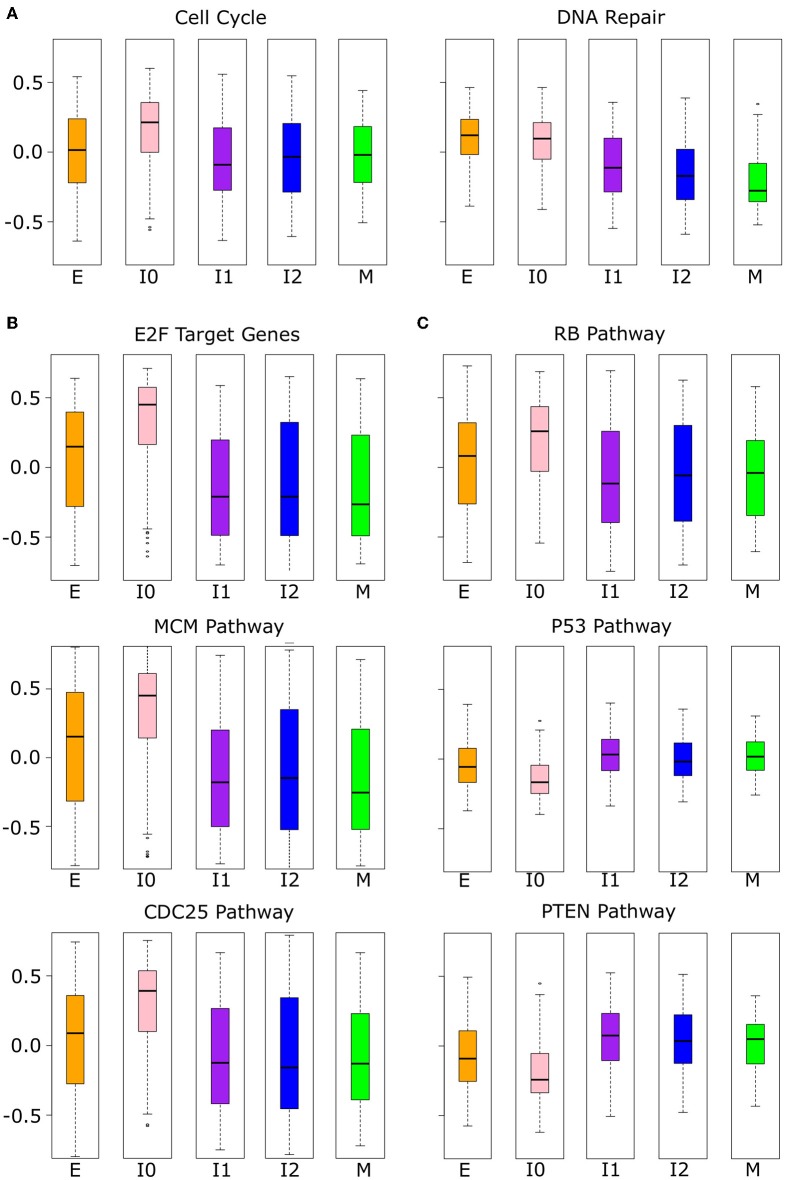
Phenotypic scores were significant in I0 BRCA cancer samples. **(A)** Boxplots showing the distribution of cell cycle and DNA repair-related gene set scores across different clusters of BRCA samples indicated by color (E = orange, I0 = pink, I1 = purple, I2 = blue, M = green). **(B)** Boxplots showing the distribution of proto-oncogene (CDC24, E2F, and MCM) pathways across different clusters of BRCA samples indicated by color as in **(A)**. **(C)** Boxplots showing the distribution of tumor suppressor (P53, PTEN, and RB) pathway scores across different clusters of BRCA samples indicated by color as in **(A)**. The *p*-values of the comparison of scores between clusters can be found in Supplementary Tables 13, 14.

While the analysis of proliferative and migratory genes implies the functional significance of the upper path of EMT, the lower path remains largely undescribed due to the similarity of I0 to E in these measures. We performed further analysis with other high-level functional gene sets, and we found that I0 samples are distinct from other EMT clusters in the expressions of cell cycle and DNA repair genes (Figure 10A). Cell cycle genes are significantly up-regulated in I0 compared to all other EMT clusters (see Supplementary Table 13) and there is a significant decrease in DNA repair associated gene expression from E to I1 (Welch's *t*-test, *p* = 4.16 × 10^−26^) that is not seen from E to I0 (Welch's *t*-test, *p* = 3.51 × 10^−1^). We next analyzed three sets of proto-oncogene pathways (E2Fs, MCM, and CDC25; Figure 10B) and three sets of tumor suppressor pathways (RB, P53, PTEN; Figure 10C) that are related to the cell cycle and cell survival. All proto-oncogene pathways showed increased expression in I0 compared to other EMT clusters, while pathways of tumor suppressors were decreased, except for RB, which also had increased expression (see Supplementary Table 14). The higher expression of RB associated genes may be due to the significant overlap in the RB pathway with those of CDC25 (66.7%) and E2F (58.3%). In general, there is a large degree of overlap between proto-oncogene pathways, but not within tumor suppressor pathways or between tumor suppressors and proto-oncogene pathways, except for RB (Supplementary Table 12). In terms of the expression of these tumor suppressors themselves, both RB and PTEN are down-regulated in I0 compared to I1, I2, and M, but not P53 (Supplementary Figure 11, Supplementary Table 15). RB and PTEN were previously shown to be mutated or down-regulated in triple-negative breast cancer (also known as basal-like) (63–66). These results further suggest that different intermediate EMT states or paths have distinct signatures that can be potentially used for diagnosis or treatment of particular cancer subtypes (67, 68).

**Figure 11:**
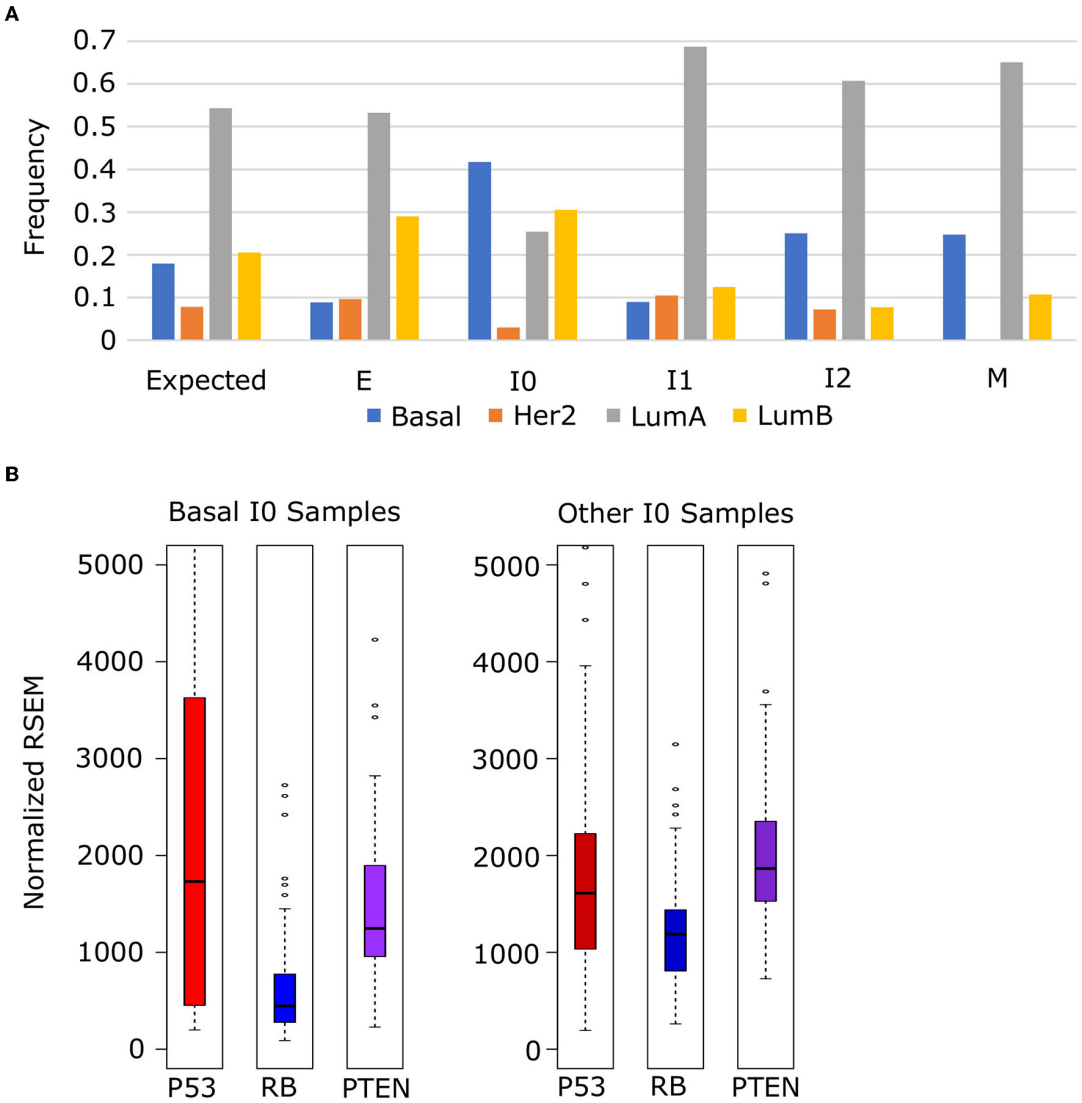
Enrichment of basal-subtype BRCA tumors in the I0 cluster. **(A)** The frequency of Luminal A (gray), Luminal B (yellow), Basal (blue), and Her2 Enriched (orange) breast cancer subtypes among breast cancer samples in each of the five EMT clusters. **(B)** The expression of tumor suppressors P53 (red), RB (blue), and PTEM (purple) in Basal I0 samples vs. all other I0 samples.

To explore the relationship between these tumor suppressors and cancer subtypes, we obtained the subtype annotation of BRCA samples from TCGA and analyzed frequency of each subtype among our EMT clusters. We used a chi-squared test to evaluate the distribution of subtypes against a null model where the frequency in each cluster matched the background distribution (Figure 11A) and found that the distribution of Luminal A (*p* = 4.05 × 10^−8^), Luminal B (*p* = 7.90 × 10^−10^), and Basal (*p* = 2.55 × 10^−19^) subtypes differed significantly across EMT clusters (HER2 Enriched was also significant, *p* = 0.72 × 10^−3^, but violated the assumptions of chi-squared test due to low counts in I0 and M). In general, we observed an increase in Her2 subtypes (Luminal A and Basal) from E to M, but Basal samples specifically are enriched in I0, which accounts for 41.5% of all I0 samples and 38.7% of all Basal samples. Compared to other I0 samples, Basal I0 samples have decreased RB (Welch's *t*-test, *p* = 1.84 × 10^−9^) and PTEN (Welch's *t*-test, *p* = 1.69 × 10^−3^) expression, but P53 is not significantly different in either direction (Welch's *t*-test, *p* = 9.68 × 10^−2^), though P53 is more variable among I0 Basal samples (Figure 11B). Taken together, these results indicate that I0 samples are enriched in the loss of clinically significant features, including both hormone receptors and tumor suppressors, though it is unclear if this is directly related to the down-regulation EMT genes in general or if the correlation arises from some linkage to additional factors.

## Discussion

The diversity of cancer cells regarding their relationship to EMT has been found in numerous previous studies (14, 21, 24, 26). However, the distribution of cancer cells in terms of the overall activities of E- and M-genes was unclear. In this study, we used integrative transcriptomic data analysis to show that cancer cells from various organs are widely distributed across the E- and M-gene expression space and that a non-linear path connecting E and M via a high-E–high-M region describes a large fraction of breast, lung, pancreas, cervical, and prostate cancer cells. With further transcriptomic analysis using non-tumorigenic cells, we found that this EMT path is consistent with the progression of TGF-β-induced EMT in normal cells over time. A previous study with breast cancer cells also showed a similar consistency in terms of a binary model between E and M states (69). These results suggest that the gene regulatory network in epithelial cells govern high-E–high-M cellular states intrinsically, and a large fraction of cancer cells show this expression pattern. Nonetheless, future experiments are warranted to demonstrate the existence of and the relationship between the cancerous intermediate EMT states and their normal counterparts. Previous mathematical models based on core EMT regulatory networks have explained the hybrid nature of partial EMT states (15–18, 70, 71). Our analyses complement these mechanistic dynamical models by demonstrating that the hybrid EMT states in terms of the overall transcriptional activity are prevalent in cancer cells. These EMT states not only show significant activities of E- and M-genes comparable to those at extreme E and M states, respectively, but are also associated with gene expression patterns corresponding to high motility and proliferation potentials, suggesting their hybrid cellular phenotypes.

Our analyses of breast cancer samples focused on a GMM with five clusters. Although this model was the best-performing model among models with one to nine clusters (see Methods), we extended our analyses to a four-cluster model and a six-cluster model (Supplementary Figures 5, 12–14). The distribution of the four clusters in the former model is clearly consistent with the non-linear EMT paths that we obtained with the five-cluster model (Supplementary Figure 5). Since it is less obvious whether the six-cluster model generates results inconsistent with our main conclusions, we performed segmented regression, analyzed the key EMT gene expression, made comparison with time course data, and examined the functional enrichment of all clusters with the six-cluster model (Supplementary Figures 12–14). All results were consistent with those obtained with the five-cluster model. Therefore, the main conclusions of this study do not depend on the exact number of clusters in the GMM.

We used statistical models to describe possible subpopulations in the cancer cell transcriptomes. It is possible that these subpopulations (clusters) of cells correspond to attractors in the EMT spectrum. In particular, both the normal EMT process and some cancer cell subpopulations show high-E–high-M expression pattern, suggesting the existence of attractors within that expression pattern. Future work is needed to relate the subpopulations in the statistical models to attractors (e.g., stable steady states) in dynamical systems, which would require a combination of transcriptomic measurement and tests of stability of the cell phenotypes. Previous work has shown the stability of a hybrid E/M phenotype in lung cancer cells (17). More systematic analysis will be needed to draw a general conclusion about the attractor property of the hybrid cancer cells in various other organs.

We found that the EMT path through a high-E–high-M region can be a major EMT path in both cancer and normal cells. This conclusion does not exclude the possibility that other EMT paths exist in significant populations of cancer cells. In fact, clusters of cells near the low-E–low-M region were found in the EMT spectrum, and they may also contribute to the transition EMT states observed in tumor cells (20). Future work involving single cell analysis is needed to reveal EMT paths in the cancer settings at higher resolutions (72–74). It is also possible that frequent transitions involving both EMT and MET, and those between intermediate states (e.g., high-E–high-M from/to low-E–low-M) occur in tumorigenesis (69, 75, 76). These transitions may be driven by paradoxical signals, such as simultaneous up-regulation or down-regulation of both EMT promoting and inhibiting factors in the microenvironment or the decoupling of the activating and repressing functions of EMT, as seen in the TES scores of the I0 and I1 clusters. The correlations between cancer and non-tumorigenic cells in the EMT spectrum do not imply the similarity of the dynamics of EMT between cancer and normal cells. In fact, genetic perturbations are likely to be a major factor contributing to the diversity of cancer cell transcriptomes. The EMT paths in the cancer samples suggest the directions of changes of cellular properties upon the gradual genetic or non-genetic perturbations to these cells. In addition, these correlations suggest that the existing regulatory pathways may channel the perturbed cells into some defined states, so long as the disruption of EMT pathways is not dramatic.

The summary scores that we used to quantify the overall activities of E- and M-genes are based on the expression of a list of EMT genes. The advantage of such metrics is that the scoring is robust to the change of individual genes. However, performing clustering on cells based on low-dimensional scores has the disadvantage of missing useful information in high-dimensional gene expression space, which has even greater potentials to reveal multiple attractors (77). This can be seen in part in the scores of E and M gene subclusters, in that the initial increase in E score during the time course appears to be driven by a specific subset of epithelial genes with distinct regulation by EMT factors. Moreover, the diversity of cancer cells must be beyond their status in the EMT spectrum (30, 78). Nonetheless, in case of proliferation and migration, we found that the activity of many functional gene sets is highly correlated with EMT status despite little or no overlap with annotated EMT genes. Overall, our clustering method is not aimed to provide a general clustering framework for analyzing cancer cells. Instead, given that the goal of our study is to find the overall patterns in the EMT spectrum for better understanding cancer cell diversity, the E- and M-gene scores serve as a concise approach to summarize the gene expression in cancer cells.

It was proposed in earlier studies that the mesenchymal state is associated with tumorigenesis (79). More recently, there was a refinement of the concept in terms of the roles of EMT in cancer progression. Multiple studies involving tumorigenesis models or analysis of tumor cells suggest the critical roles of partial EMT in metastatic processes (3, 80, 81). In addition, cellular functions of both epithelial and mesenchymal genes contribute to the formation of secondary tumors (7, 81). This suggests that metastasis may involve synergy among various cell types in multiple positions on the EMT spectrum. Our work further suggests that a significant population of cancer cells possess hybrid functions. Although it is likely that tumor formation requires interactions of multiple types of cells, the multifunctional nature of the hybrid cells, in particular their potential to migrate and proliferate, might be an important factor contributing to the invasiveness of cancer cells.

## Data Availability Statement

The results shown here are in whole or part based upon data generated by the TCGA Research Network: https://www.cancer.gov/tcga. All datasets generated for this study are included in the article/Supplementary Material. All computer code for data analysis is available upon request.

## Author Contributions

NP, CA-T, ML, NR, and TH performed the research. NP and TH wrote the manuscript. TH conceived the study. All authors have read and approved the final manuscript.

### Conflict of Interest

The authors declare that the research was conducted in the absence of any commercial or financial relationships that could be construed as a potential conflict of interest.

## Supplementary Material

Supplementary Figure 1 Graphs of the Bayesian Information Criterion (BIC) against the number of clusters in the model of BRCA samples. **(A)** The BIC of six different variance model, indicated by the color the line (EII = red, VII = orange, EEI = green, VEI = blue, EVI = purple, VVI = pink). **(B)** The average BIC across all six models. Note that, in both graphs, the BIC of the five cluster models have the minimum value and occurs at the cusp of the graph, where additional cluster cease to produce large reduction in BIC.

Supplementary Figure 2 Clustering of LUAD transcriptome samples using different variance models. Note the difference between models with equal volume (top) and varying volume (bottom).

Supplementary Figure 3 Histogram of uncertainty (1—max probability of cluster membership) for different GMM models of LUAD transcriptome samples using different variance models. Note decreased uncertain for models with equal volume (top) compared to those with varying volume (bottom).

Supplementary Figure 4 Migration score for RUNX2 pathway in BRCA samples including only cancer samples (left) and all samples (right). Note the decrease in proliferation scores for M and I2 when normal samples are included as most of these samples are clustered with M or I2. This suggest that these samples are functional different from cancer samples and therefore were excluded from further functional analysis.

Supplementary Figure 5 Clustering of BRCA samples by GMM using four **(A)** and six **(B)** clusters. Individual samples are indicated by points in E-M score space with their assigned cluster indicated by color (E = orange, I0 = pink, I1 = purple, I1' = blue, I2 = green, M = red). Contour lines indicate the predicted distribution of the underlying models. Black dots denote the center of each Gaussian distribution. Note that the major difference in the four clusters model is that the most extreme low E, high M cluster now include the majority (69%) of I2 samples from five-cluster GMM, the remainder being split between I1 and I0. Similarly, in the six-cluster GMM model, I1 samples from the five-cluster GMM model are divided into two clusters, I1 and I1', with I1' consisting of samples with the most extreme E and M expression.

Supplementary Figure 6 Contour plots of LUAD (top-left), PAAD (top-right), CESC (bottom-left), and PRAD (bottom-right) samples in E-M score space.

Supplementary Figure 7 GMM models of LUAD (top-left), PAAD (top-right), CESC (bottom-left), and PRAD (bottom-right) samples in E-M score space. Individual samples are indicated by points in E-M score space with their assigned cluster indicated by color which roughly correspond to the clusters assigned to BRCA but for the absence of an I2 intermediate state (E = orange, I0 = pink, I1 = purple, M = green).

Supplementary Figure 8 Segmented models E-M score relationship among LUAD (top-left), PAAD (top-right), CESC (bottom-left), and PRAD (bottom-right) samples. Three models are shown: one based on all samples (black line), one excluding I0 samples (upper path, light red line), and on excluding I1 samples (light blue lines). Individual samples are shown by points with the color of the point corresponding to the whether the point is unique the upper path (blue), lower path (red), or common to both paths (purple).

Supplementary Figure 9 Probabilities of cluster membership across segmented models of E-M scores in the TGF-β induced EMT time course. Three segmented models are evaluated, the best fit and the upper and lowers bounds, which are based on the confidence interval of the time of the first breakpoint in the relationship between time after TGF-β induction and E-score. Probabilities are estimated by evaluating the segmented model every 0.25 days from 0 to 21 days and clustering the resulting E-M scores using the BRCA GMM model.

Supplementary Figure 10 The response of subculsters of epithelial (E1, E2, E3) and mesenchymal (M1, M2, M3) genes to TGF-β and ZEB1. Each boxplot shows the distribution of fold change of expression to eight contrast conditions between TGF-β induction (TGFβ) ZEB1 induction (DOX), TGF-β inhibition (SB), and ZEB1 inhibition (dZEB) and their respective controls (WT for TGF-β induction and DMSO for ZEB1 induction). Note that, for M3 genes, the distribution of fold change is highest for DOX vs. DMSO and DOX + SB vs. SB, indicating increased expression in response to ZEB1 regardless of TGF-β expression and that, for E2 genes, the distribution of TGF-β + dZEB vs. dZEb is most positive, indicating that, when dZEB is absent or repressed, TGF-β can induced expression of these epithelial genes. Following the methodology applied to M-genes in (23), the regulation of E-gene subclusters was inferred from their response to each condition. The response of E1 to each factor is independent of the other factor (compare TGF-β vs. WT to TGF-β + dZEB vs. dZEB and DOX vs. DSMO to DOX + SB vs. SB), similar to M2, so we infer regulation occurs via an “OR” gate. E3 genes are repressed by expression TGF-β in the presence of ZEB1 (TGF-β vs. WT), but unaffected by ZEB1 in the presence (DOX vs. DMSO) or absence (DOX + SB vs. SB) of TGF-β. As such, E3 regulation is similar to M1, requiring integration of TGF-β and ZEB1 via an “AND” style logic gate. However, unlike M1, the absence of ZEB1 doesn't suppress the TGF-β effect on E3. Rather, TGF-β has the opposite effect on E3 genes without ZEB1 (TGF-β + dZEB vs. dZEb). Finally, E2 genes are repressed by ZEB1 (DOX vs. DMSO), independent of TGF-β (DOX + SB vs. SB), while TGF-β activates E2 (TGF-β + dZEB vs. dZEB), though this effect is suppressed in the absence of ZEB1 (TGF-β vs. WT). While this pattern is difficult to describe using a single logical gate, it is easy to understand as ZEB1 regulating E2 both directly and by suppressing the activating function of ZEB1. Therefore, E2 is similar to M3 in that ZEB1 plays the dominant role in regulating the expression.

Supplementary Figure 11 Boxplots showing the distribution of P53, PTEN, and RB expression across different clusters of BRCA samples indicated by color (E = orange, I0 = pink, I1 = purple, I2 = blue, M = green). A full table of *p*-values can be found in Supplementary Table 15.

Supplementary Figure 12 TGF-β induced EMT time course in E-M score space with six GMM clusters. **(A)** Segmented models of the relationship between the time since TGF-β induction of EMT and the E-M scores of treated cells. Different colored lines correspond to the best fit (black), lower bound (light red), and upper bound (light blue) models. Individual BRCA samples are indicated as by open points with their color corresponding to the assigned cluster (E = orange, I0 = pink, I1 = purple, I1' = blue, I2 = green, M = red). Note that, much like the 5-cluster GMM models, the models pass from E to I1 to I2 to M, with only the upper bound intersecting with I1'. **(B)** The predicated probability of samples at a point in segmented model TGF-β induced EMT belonging to each cell state cluster from the six cluster GMM model of BRCA samples. The probabilities were calculated by applying the BRCA GMM model to segmented model of E-M scores with respect to time after TGF-β induction. Note that I1' appears for a small period of time between I1 and I2 and never accounts for the majority predictions of the cell state at any point during TGF-β induced EMT.

Supplementary Figure 13 Boxplots showing the distribution of ZEB1 expression across six clusters of BRCA samples indicated by color (E = orange, I0 = pink, I1 = purple, I1' = blue, I2 = green, M = red). As with the five cluster GMM model of BRCA samples, the differences between E-I1-I2-M clusters are significant. The I1' cluster has significantly higher expression than I1 and significantly lower expression than M, but is not different from I2. Expression values are normalized RSEM (RNA-Seq by Expectation Maximization) values.

Supplementary Figure 14 Phenotypic scores BRCA cancer samples in the six cluster GMM model. **(A)** Boxplots showing the distribution of migration related gene set scores across different clusters of BRCA samples indicated by color (E = orange, I0 = pink, I1 = purple, I2 = blue, M = green). **(B)** Boxplots showing the distribution of proliferation related gene set scores across different clusters of BRCA samples indicated by color as in **(A)**. Note that in both cases, the association between BRCA clusters and phenotypic data is the same, with proliferation being maintained through I1 and migration increasing at I1, though I1 occasionally represents a peak of migration at or above the level of M.

Supplementary Table 1 BIC Scores for BRCA sample models.

Supplementary Table 2 BIC Scores for PAAD sample models.

Supplementary Table 3 BIC Scores for LUAD sample models.

Supplementary Table 4 BIC Scores for CESC sample models.

Supplementary Table 5 BIC Scores for PRAD sample models.

Supplementary Table 6 Adjusted R-squared values for different breakpoint models.

Supplementary Table 7 Description of phenotypic gene sets from the Broad Institute.

Supplementary Table 8 *P*-values and 95% CI values for TES scores between clusters.

Supplementary Table 9 *P*-values for EMT Factor expression between clusters.

Supplementary Table 10 *P*-values for migration scores between clusters.

Supplementary Table 11 *P*-values for proliferation scores between clusters.

Supplementary Table 12 Overlap between E-M gene sets and Broad Institute gene sets.

Supplementary Table 13 *P*-values for cell cycle and DNA repair scores between clusters.

Supplementary Table 14 *P*-values for proto onco-gene and tumor suppressor pathway scores between clusters.

Supplementary Table 15 *P*-values for tumor suppressor gene expression between clusters.

Supplementary File 1 EMT related scores and cluster classifications for samples in TCGA.

Supplementary File 2 EMT related scores for EMT time course data.
