# Supplementary figures and images for: Integrative Transcriptomic Analysis Reveals a Multiphasic Epithelial–Mesenchymal Spectrum in Cancer and Non-tumorigenic Cells
Source: Front Oncol. 2020 Jan 22;9:1479. doi: 10.3389/fonc.2019.01479 (PMC6987415; doi:10.3389/fonc.2019.01479)

**A**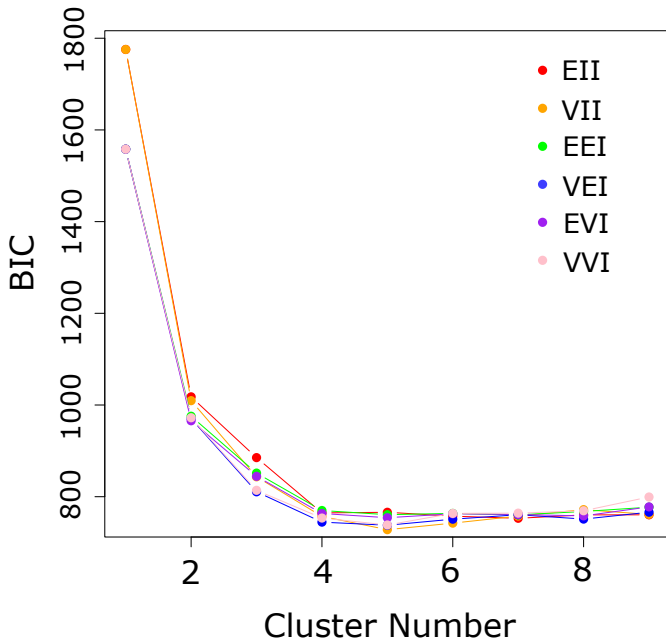**B**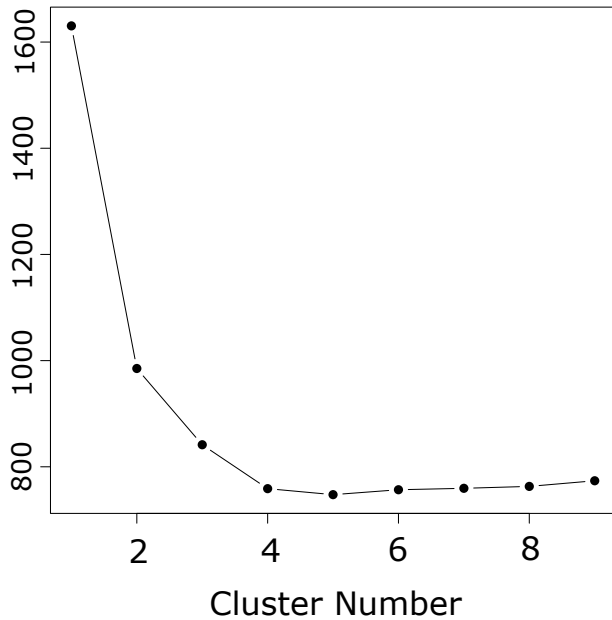

Supplement: Supplementary Figure 1 — Graphs of the Bayesian Information Criterion (BIC) against the number of clusters in the model of BRCA samples. (A) The BIC of six different variance model, indicated by the color the line (EII = red, VII = orange, EEI = green, VEI = blue, EVI = purple, VVI = pink). (B) The average BIC across all six models. Note that, in both graphs, the BIC of the five cluster models have the minimum value and occurs at the cusp of the graph, where additional cluster cease to produce large reduction in BIC. [file Presentation_1.zip › presentation 1/Supplementary Figure S1.pdf]

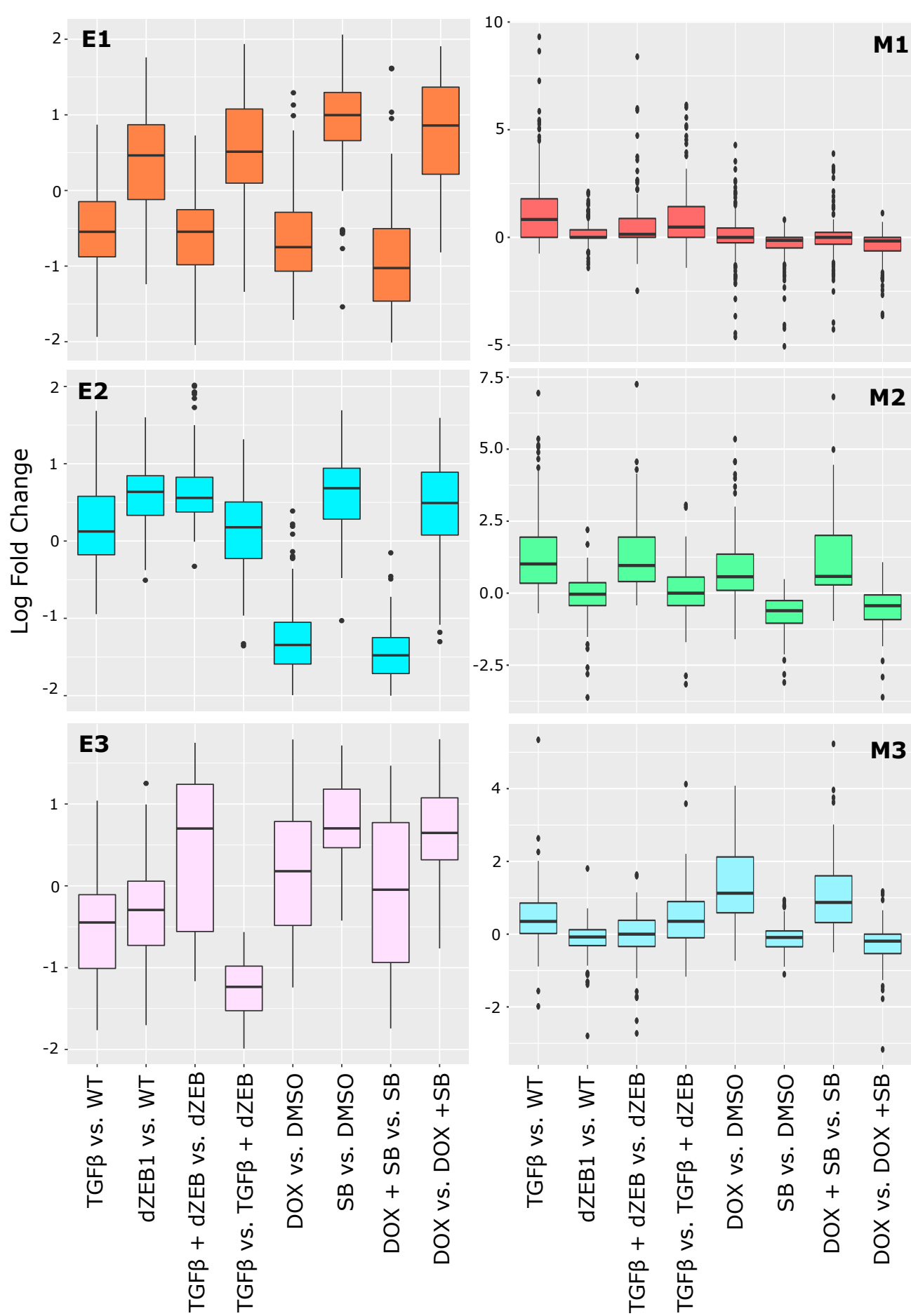

Supplement: Supplementary Figure 1 — Graphs of the Bayesian Information Criterion (BIC) against the number of clusters in the model of BRCA samples. (A) The BIC of six different variance model, indicated by the color the line (EII = red, VII = orange, EEI = green, VEI = blue, EVI = purple, VVI = pink). (B) The average BIC across all six models. Note that, in both graphs, the BIC of the five cluster models have the minimum value and occurs at the cusp of the graph, where additional cluster cease to produce large reduction in BIC. [file Presentation_1.zip › presentation 1/Supplementary Figure S10.pdf]

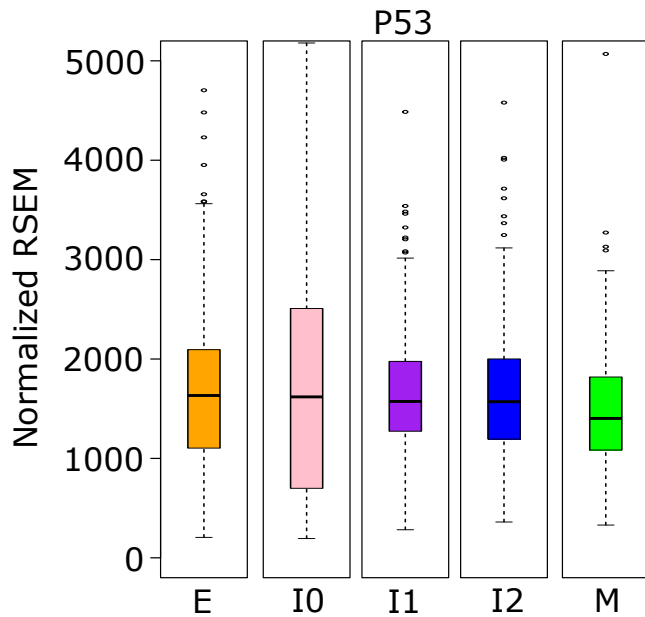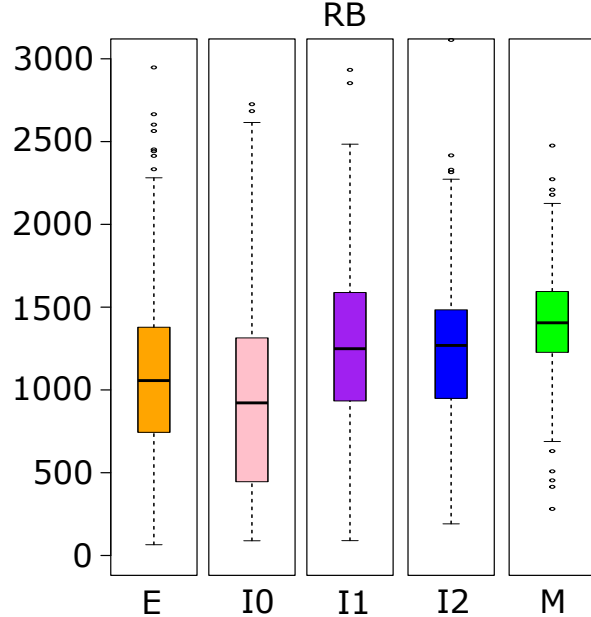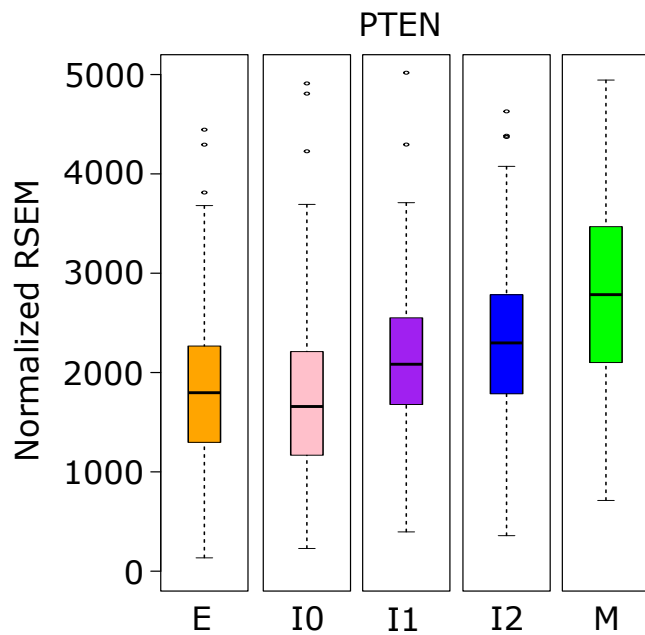

Supplement: Supplementary Figure 1 — Graphs of the Bayesian Information Criterion (BIC) against the number of clusters in the model of BRCA samples. (A) The BIC of six different variance model, indicated by the color the line (EII = red, VII = orange, EEI = green, VEI = blue, EVI = purple, VVI = pink). (B) The average BIC across all six models. Note that, in both graphs, the BIC of the five cluster models have the minimum value and occurs at the cusp of the graph, where additional cluster cease to produce large reduction in BIC. [file Presentation_1.zip › presentation 1/Supplementary Figure S11.pdf]

**A**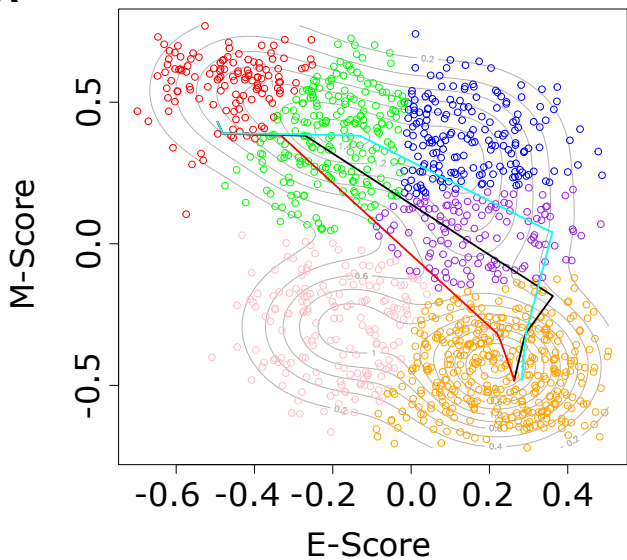**B**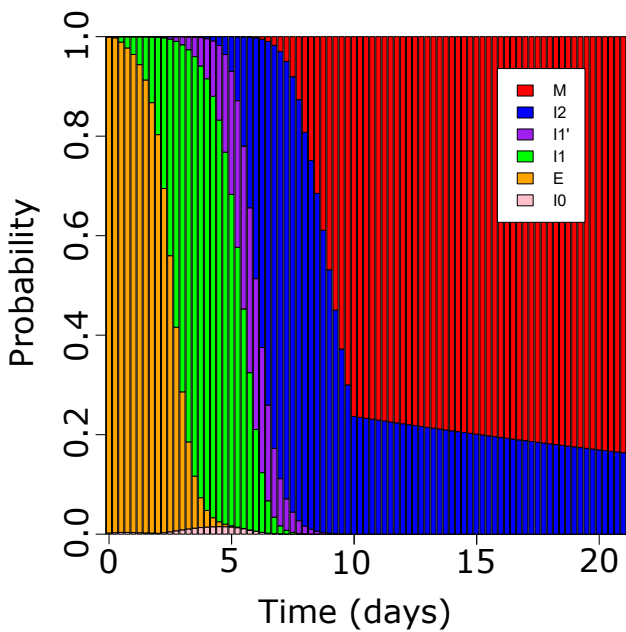

Supplement: Supplementary Figure 1 — Graphs of the Bayesian Information Criterion (BIC) against the number of clusters in the model of BRCA samples. (A) The BIC of six different variance model, indicated by the color the line (EII = red, VII = orange, EEI = green, VEI = blue, EVI = purple, VVI = pink). (B) The average BIC across all six models. Note that, in both graphs, the BIC of the five cluster models have the minimum value and occurs at the cusp of the graph, where additional cluster cease to produce large reduction in BIC. [file Presentation_1.zip › presentation 1/Supplementary Figure S12.pdf]

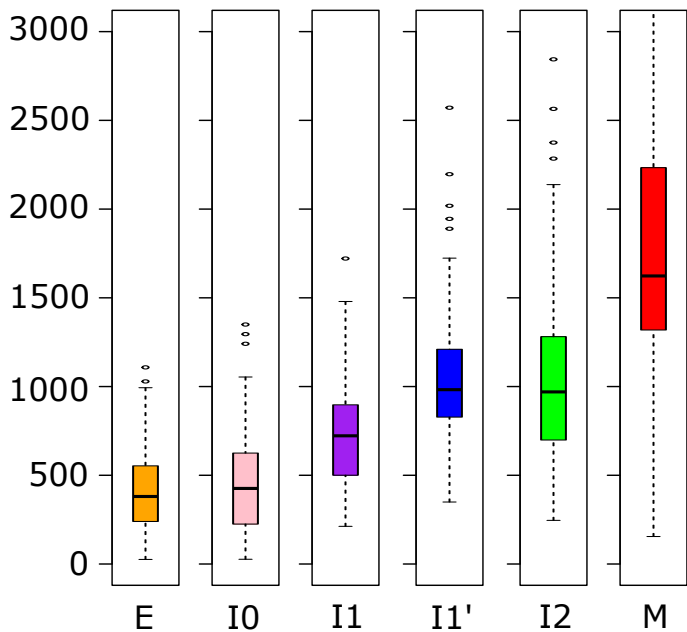

Supplement: Supplementary Figure 1 — Graphs of the Bayesian Information Criterion (BIC) against the number of clusters in the model of BRCA samples. (A) The BIC of six different variance model, indicated by the color the line (EII = red, VII = orange, EEI = green, VEI = blue, EVI = purple, VVI = pink). (B) The average BIC across all six models. Note that, in both graphs, the BIC of the five cluster models have the minimum value and occurs at the cusp of the graph, where additional cluster cease to produce large reduction in BIC. [file Presentation_1.zip › presentation 1/Supplementary Figure S13.pdf]

EII

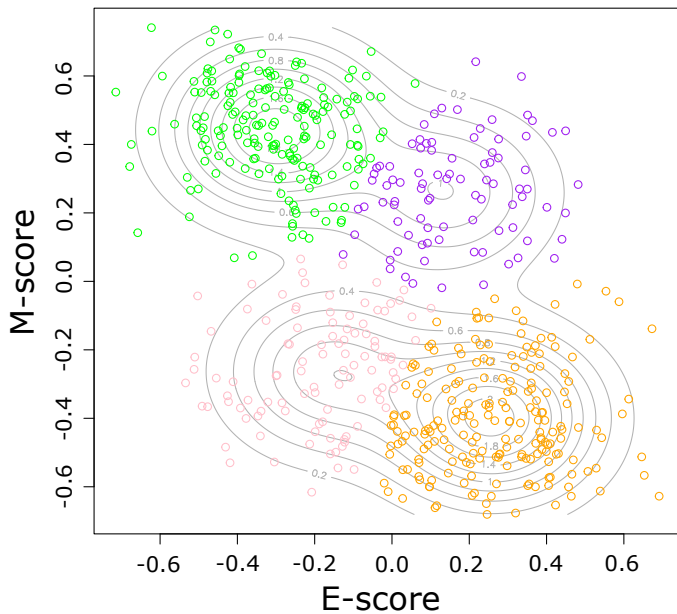

EEI

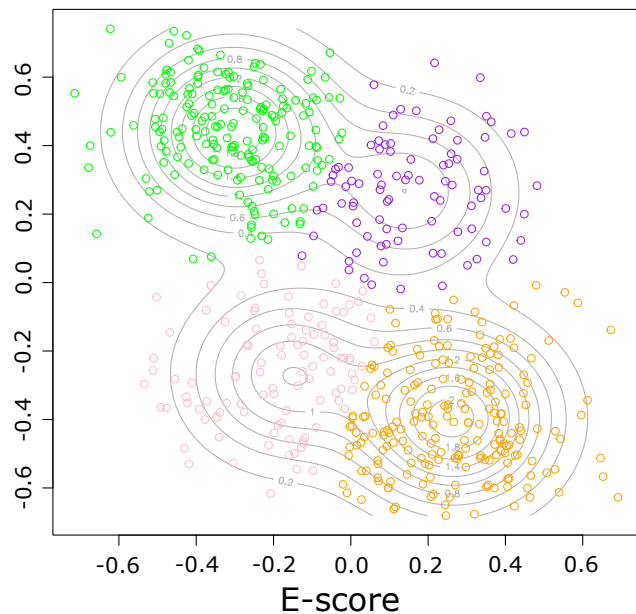

VEI

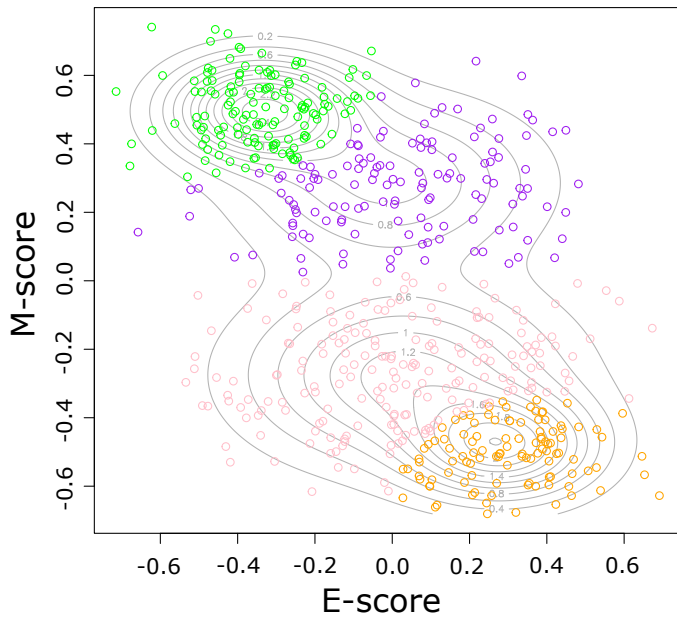

VVI

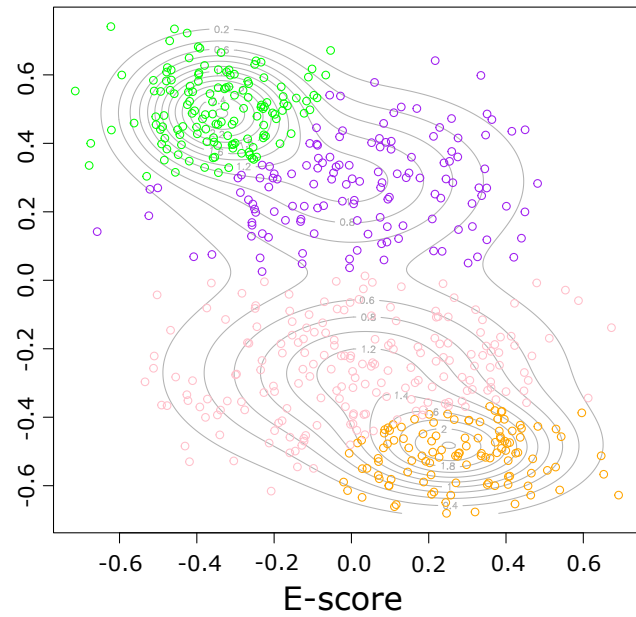

Supplement: Supplementary Figure 1 — Graphs of the Bayesian Information Criterion (BIC) against the number of clusters in the model of BRCA samples. (A) The BIC of six different variance model, indicated by the color the line (EII = red, VII = orange, EEI = green, VEI = blue, EVI = purple, VVI = pink). (B) The average BIC across all six models. Note that, in both graphs, the BIC of the five cluster models have the minimum value and occurs at the cusp of the graph, where additional cluster cease to produce large reduction in BIC. [file Presentation_1.zip › presentation 1/Supplementary Figure S2.pdf]

EII

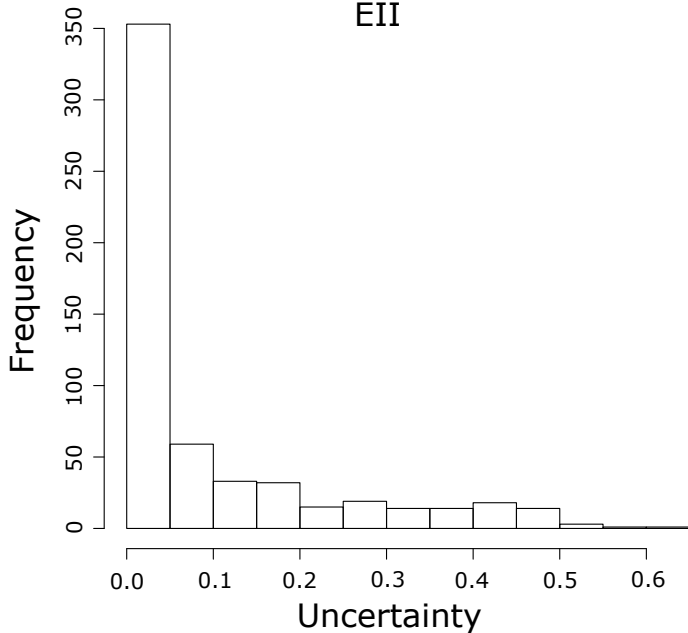

EEI

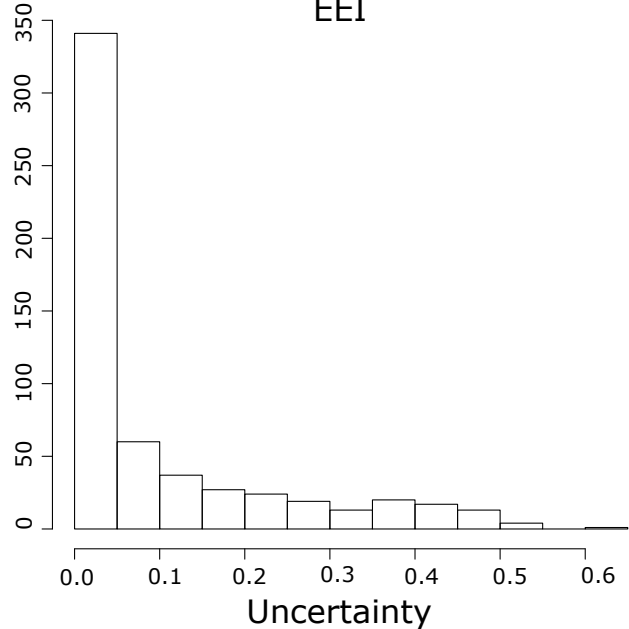

VEI

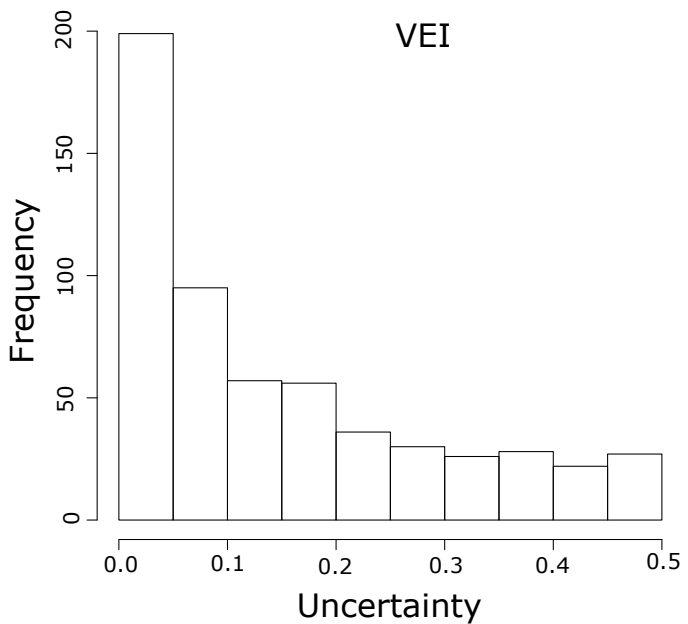

VVI

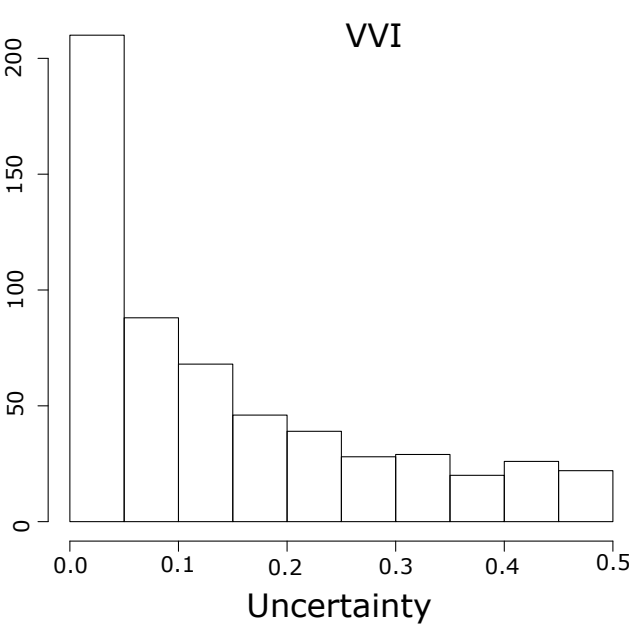

Supplement: Supplementary Figure 1 — Graphs of the Bayesian Information Criterion (BIC) against the number of clusters in the model of BRCA samples. (A) The BIC of six different variance model, indicated by the color the line (EII = red, VII = orange, EEI = green, VEI = blue, EVI = purple, VVI = pink). (B) The average BIC across all six models. Note that, in both graphs, the BIC of the five cluster models have the minimum value and occurs at the cusp of the graph, where additional cluster cease to produce large reduction in BIC. [file Presentation_1.zip › presentation 1/Supplementary Figure S3.pdf]

# RUNX2 Pathway

## Cancer Samples

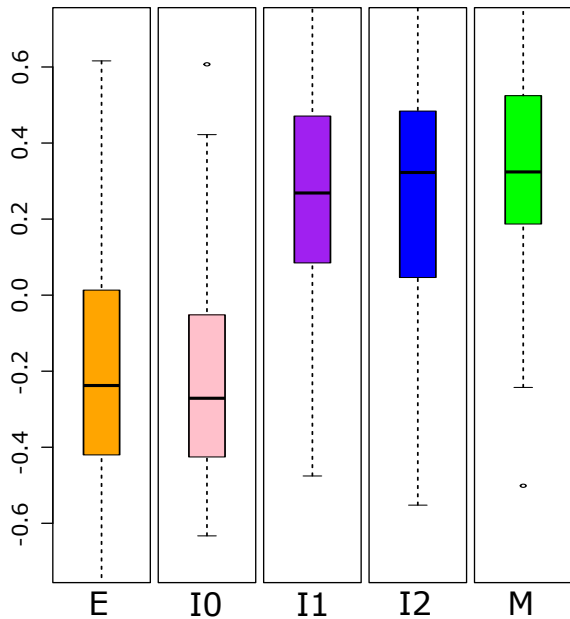

## All Samples

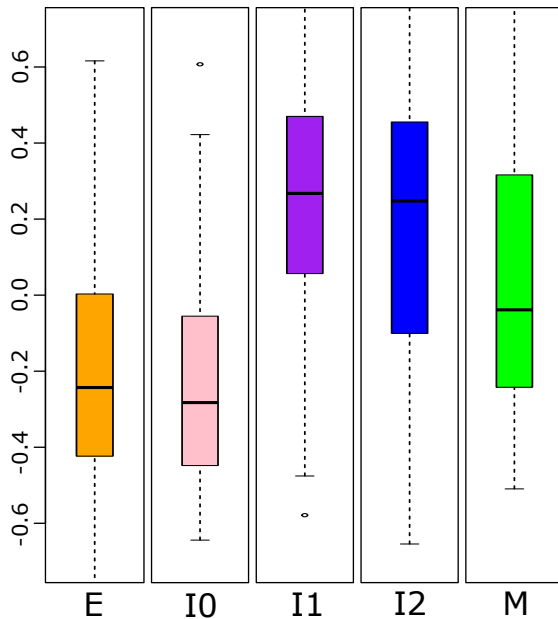

Supplement: Supplementary Figure 1 — Graphs of the Bayesian Information Criterion (BIC) against the number of clusters in the model of BRCA samples. (A) The BIC of six different variance model, indicated by the color the line (EII = red, VII = orange, EEI = green, VEI = blue, EVI = purple, VVI = pink). (B) The average BIC across all six models. Note that, in both graphs, the BIC of the five cluster models have the minimum value and occurs at the cusp of the graph, where additional cluster cease to produce large reduction in BIC. [file Presentation_1.zip › presentation 1/Supplementary Figure S4.pdf]

**A**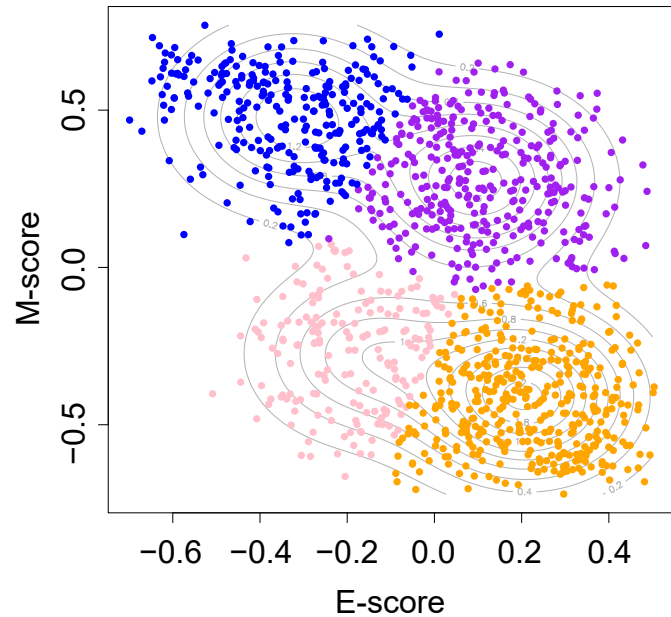**B**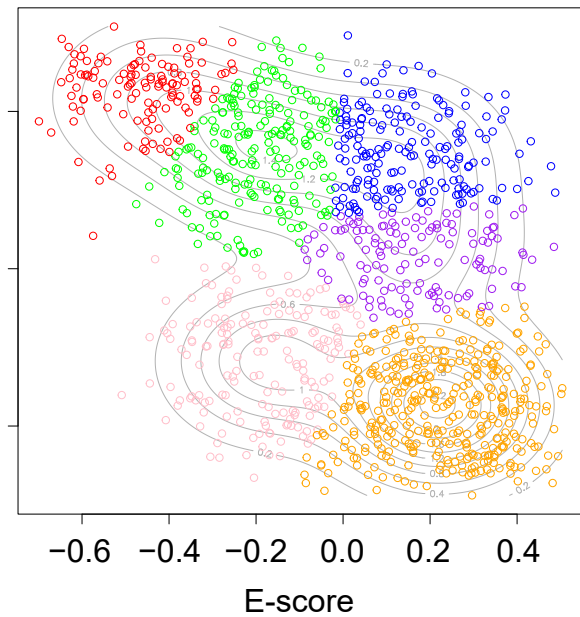

Supplement: Supplementary Figure 1 — Graphs of the Bayesian Information Criterion (BIC) against the number of clusters in the model of BRCA samples. (A) The BIC of six different variance model, indicated by the color the line (EII = red, VII = orange, EEI = green, VEI = blue, EVI = purple, VVI = pink). (B) The average BIC across all six models. Note that, in both graphs, the BIC of the five cluster models have the minimum value and occurs at the cusp of the graph, where additional cluster cease to produce large reduction in BIC. [file Presentation_1.zip › presentation 1/Supplementary Figure S5.pdf]

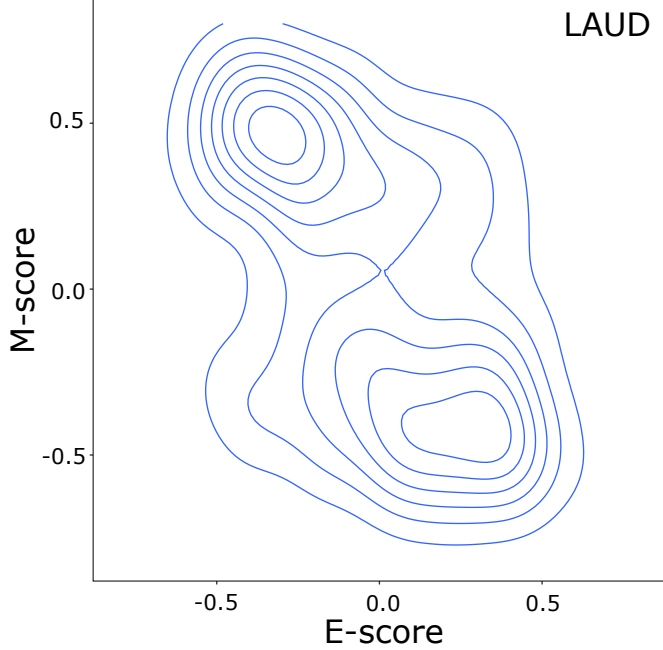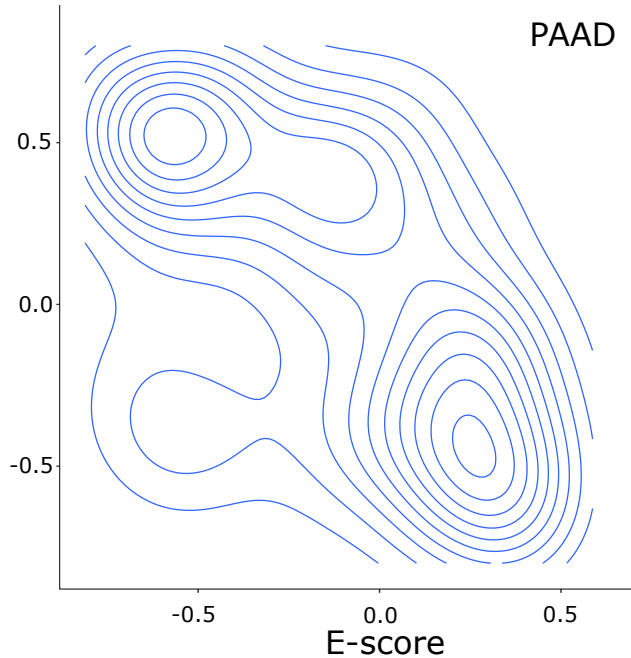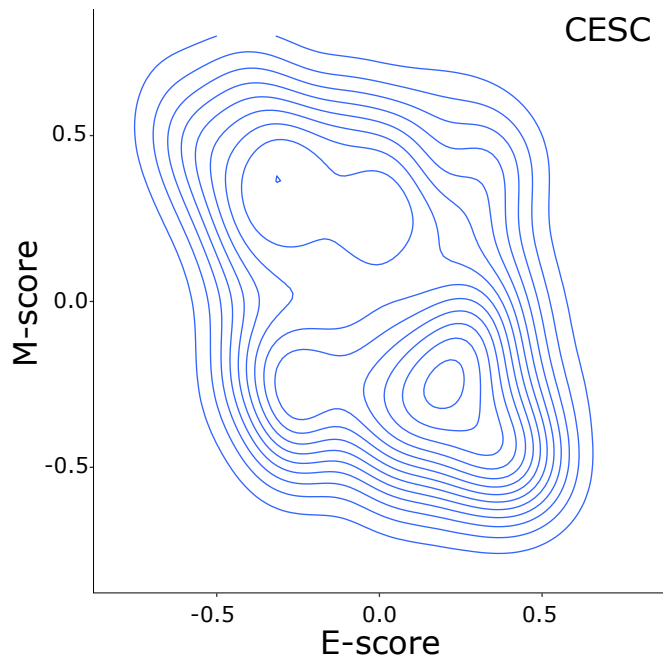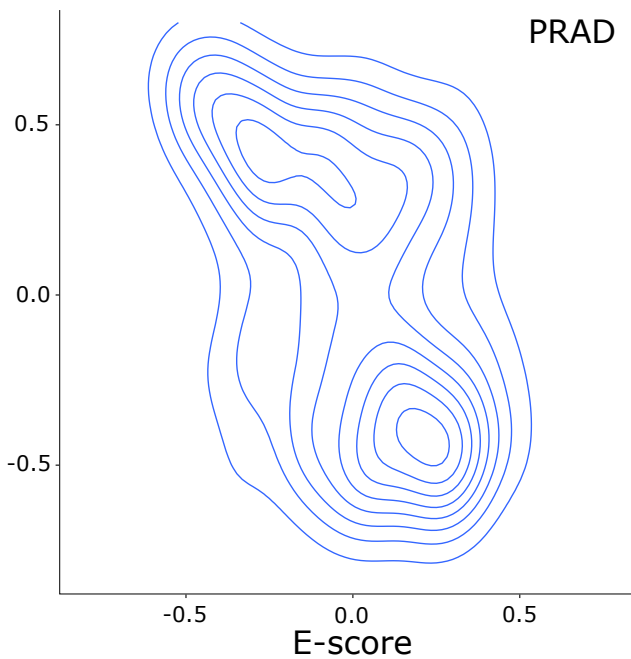

Supplement: Supplementary Figure 1 — Graphs of the Bayesian Information Criterion (BIC) against the number of clusters in the model of BRCA samples. (A) The BIC of six different variance model, indicated by the color the line (EII = red, VII = orange, EEI = green, VEI = blue, EVI = purple, VVI = pink). (B) The average BIC across all six models. Note that, in both graphs, the BIC of the five cluster models have the minimum value and occurs at the cusp of the graph, where additional cluster cease to produce large reduction in BIC. [file Presentation_1.zip › presentation 1/Supplementary Figure S6.pdf]

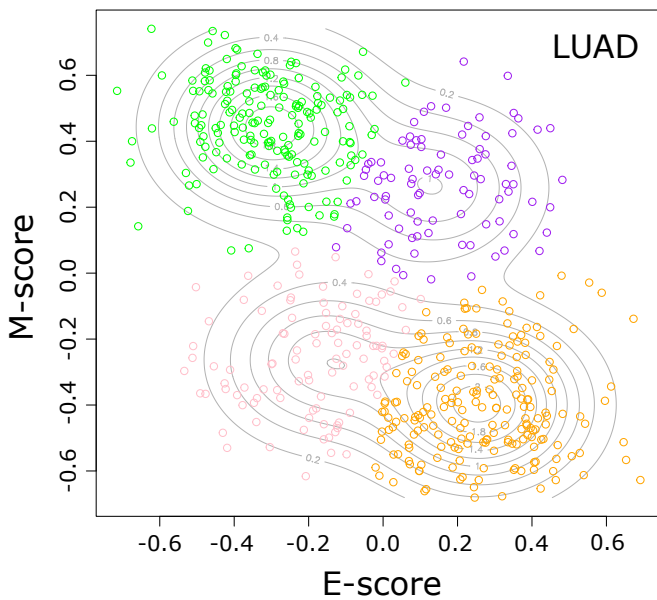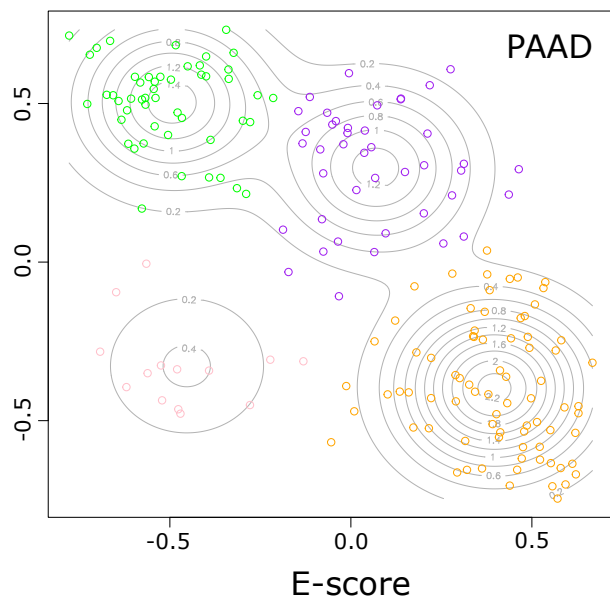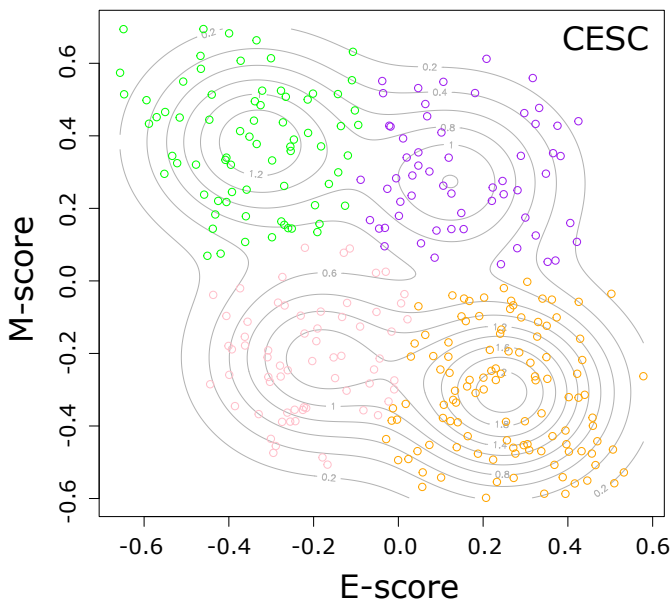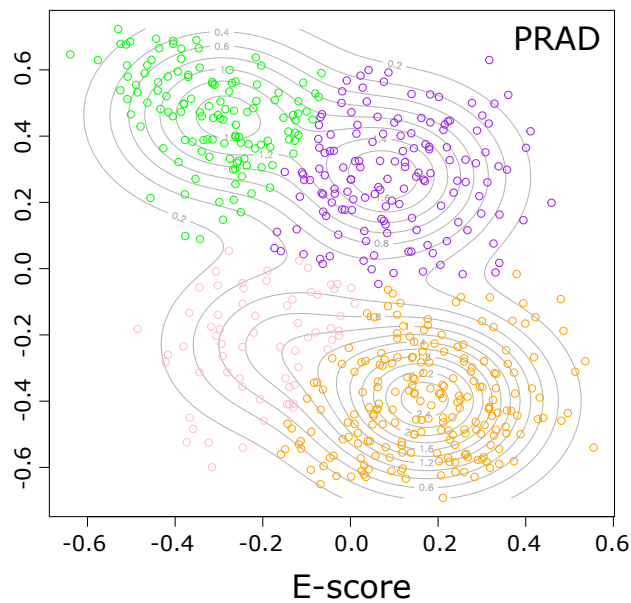

Supplement: Supplementary Figure 1 — Graphs of the Bayesian Information Criterion (BIC) against the number of clusters in the model of BRCA samples. (A) The BIC of six different variance model, indicated by the color the line (EII = red, VII = orange, EEI = green, VEI = blue, EVI = purple, VVI = pink). (B) The average BIC across all six models. Note that, in both graphs, the BIC of the five cluster models have the minimum value and occurs at the cusp of the graph, where additional cluster cease to produce large reduction in BIC. [file Presentation_1.zip › presentation 1/Supplementary Figure S7.pdf]

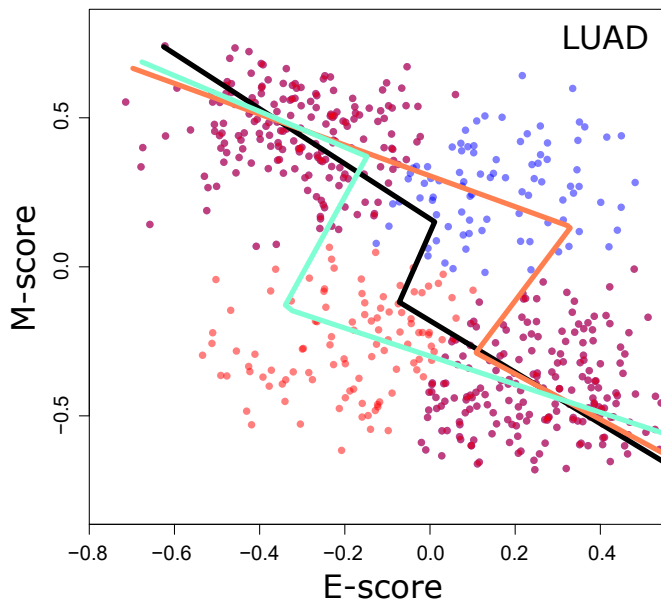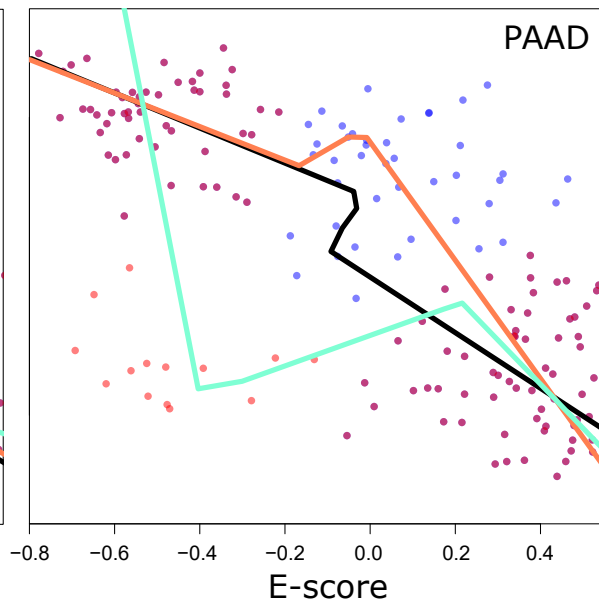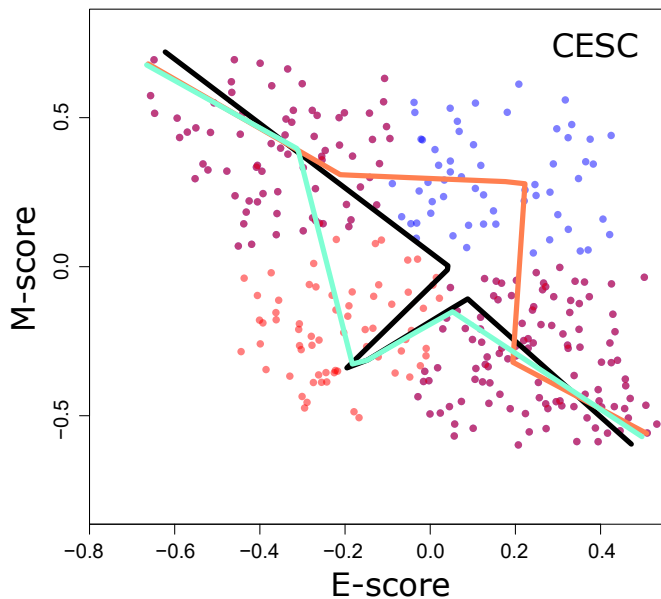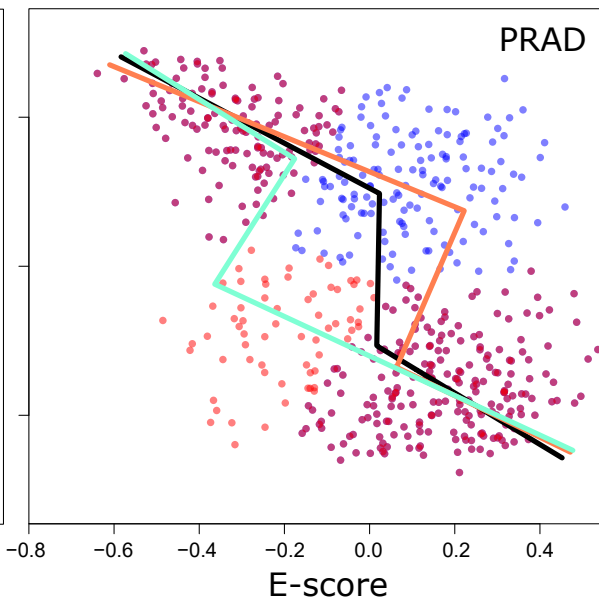

Supplement: Supplementary Figure 1 — Graphs of the Bayesian Information Criterion (BIC) against the number of clusters in the model of BRCA samples. (A) The BIC of six different variance model, indicated by the color the line (EII = red, VII = orange, EEI = green, VEI = blue, EVI = purple, VVI = pink). (B) The average BIC across all six models. Note that, in both graphs, the BIC of the five cluster models have the minimum value and occurs at the cusp of the graph, where additional cluster cease to produce large reduction in BIC. [file Presentation_1.zip › presentation 1/Supplementary Figure S8.pdf]

Time-course Model (Best-fit)

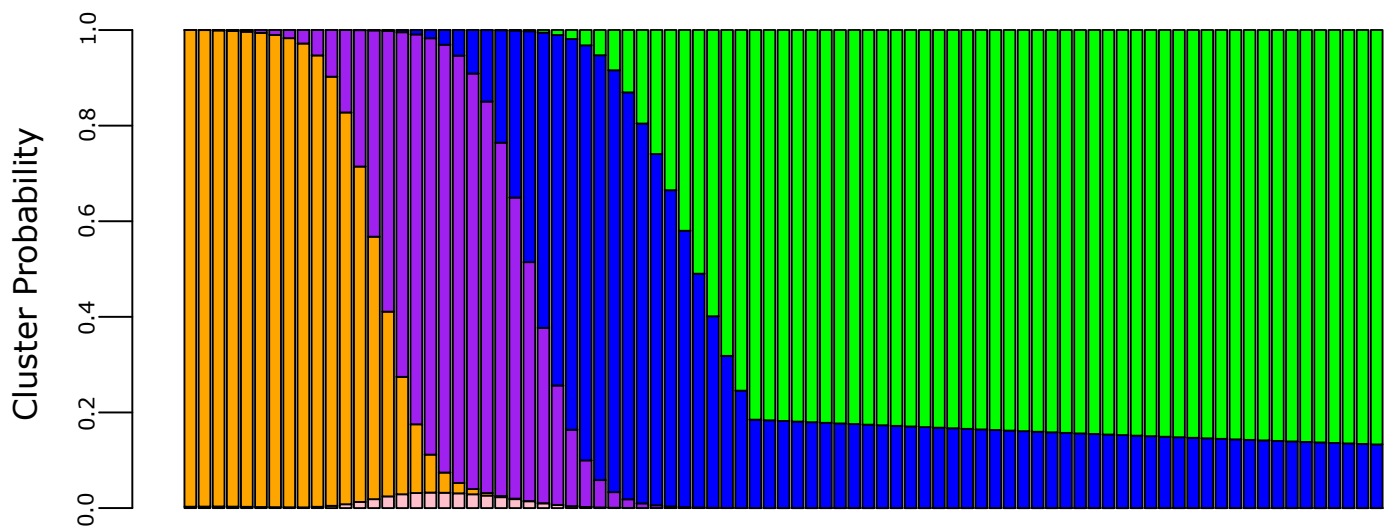

Time-course Model (Lower-bound)

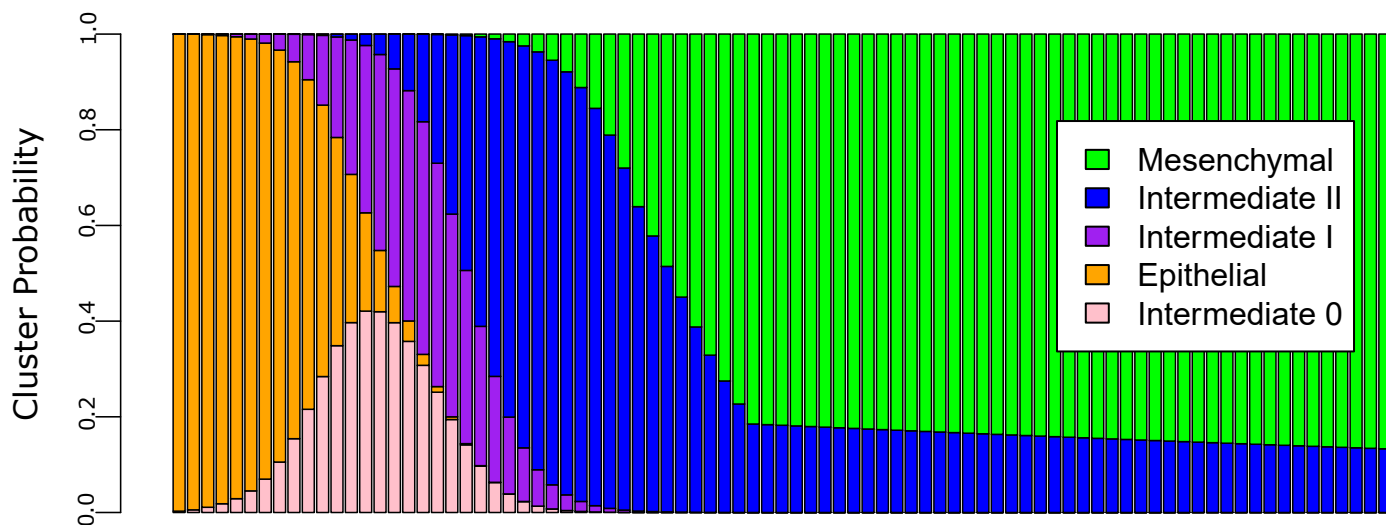

Time-course Model (Upper-bound)

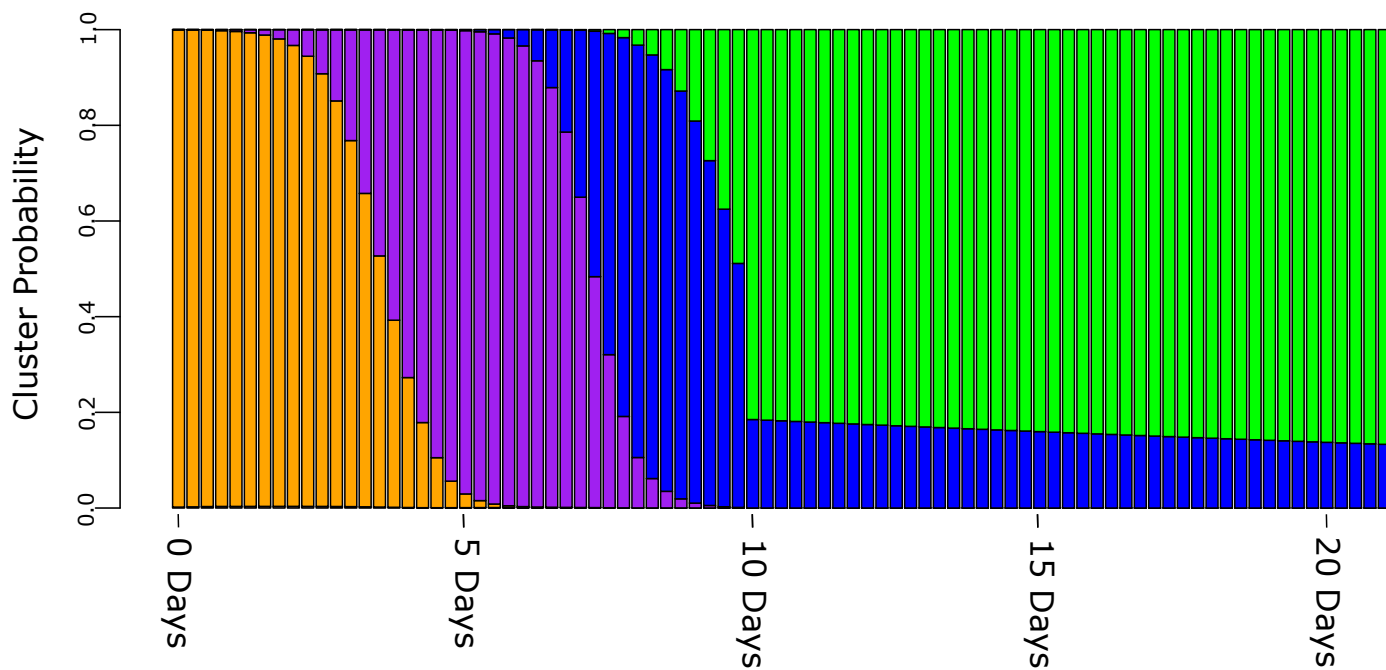

Supplement: Supplementary Figure 1 — Graphs of the Bayesian Information Criterion (BIC) against the number of clusters in the model of BRCA samples. (A) The BIC of six different variance model, indicated by the color the line (EII = red, VII = orange, EEI = green, VEI = blue, EVI = purple, VVI = pink). (B) The average BIC across all six models. Note that, in both graphs, the BIC of the five cluster models have the minimum value and occurs at the cusp of the graph, where additional cluster cease to produce large reduction in BIC. [file Presentation_1.zip › presentation 1/Supplementary Figure S9.pdf]

**A**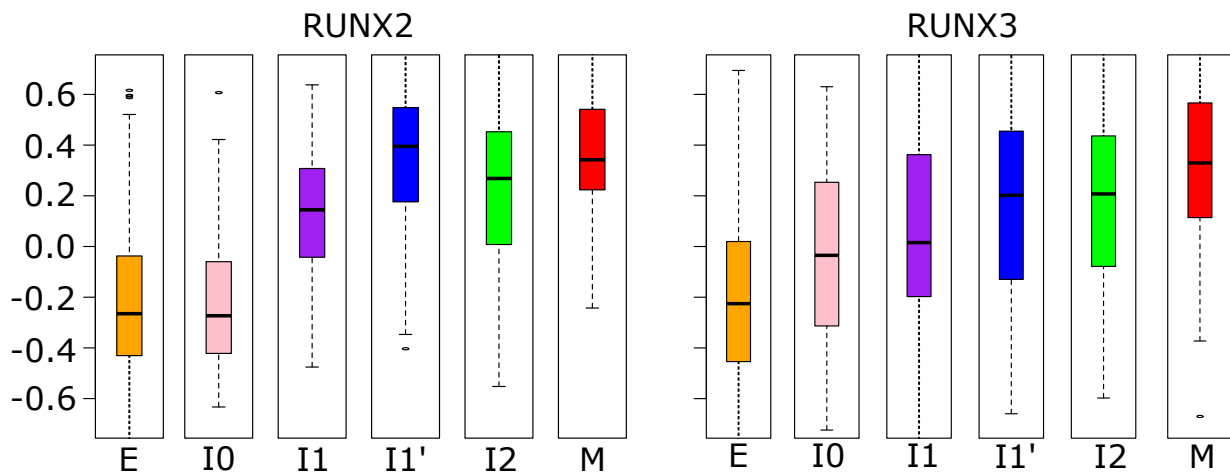**B**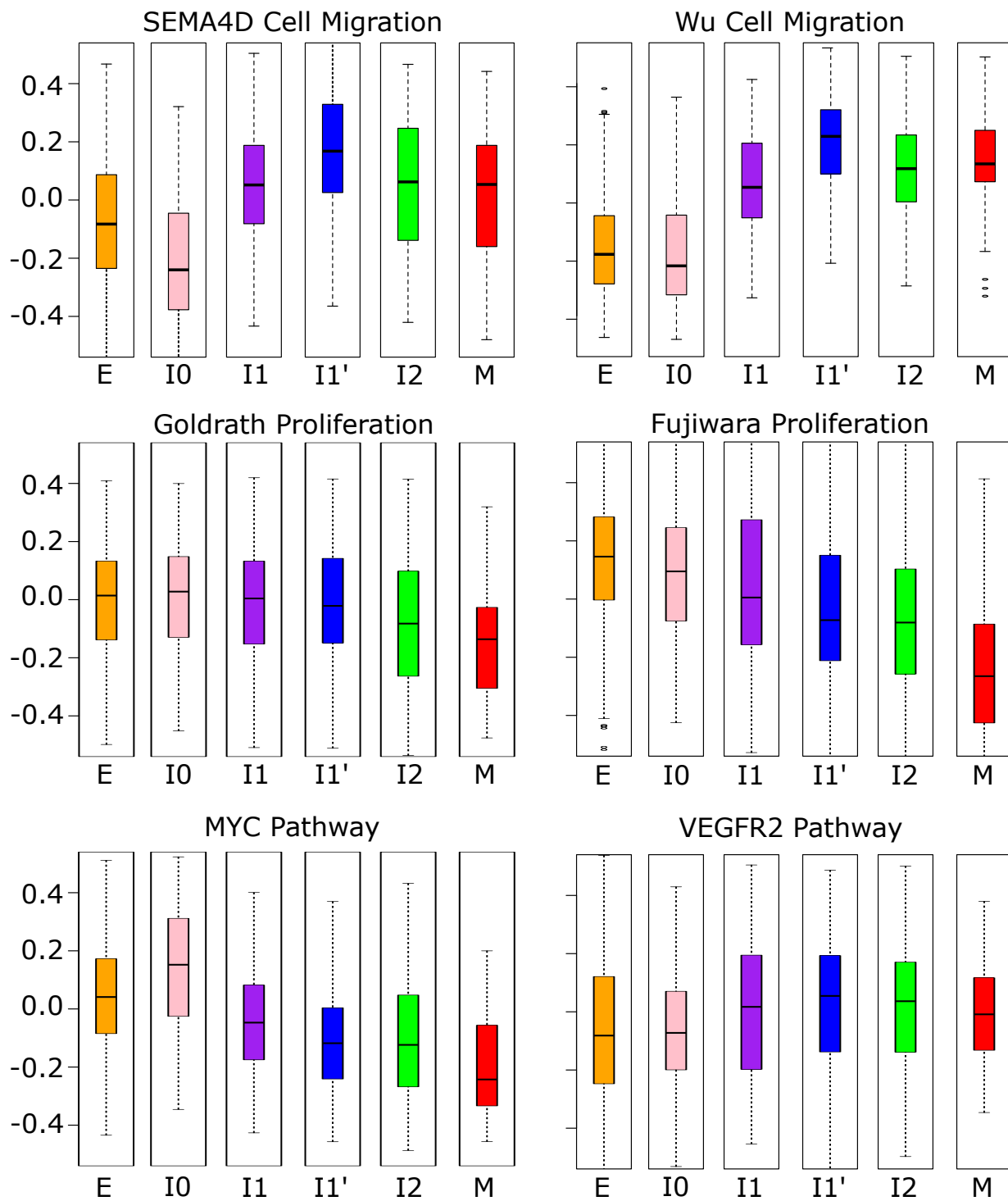

Supplement: Supplementary Figure 1 — Graphs of the Bayesian Information Criterion (BIC) against the number of clusters in the model of BRCA samples. (A) The BIC of six different variance model, indicated by the color the line (EII = red, VII = orange, EEI = green, VEI = blue, EVI = purple, VVI = pink). (B) The average BIC across all six models. Note that, in both graphs, the BIC of the five cluster models have the minimum value and occurs at the cusp of the graph, where additional cluster cease to produce large reduction in BIC. [file Presentation_1.zip › presentation 1/Supplemetnary Figure S14.pdf]
